# Aging-related elevation of sphingoid bases shortens yeast chronological life span by compromising mitochondrial function

**DOI:** 10.18632/oncotarget.8195

**Published:** 2016-03-19

**Authors:** Jae Kyo Yi, Ruijuan Xu, Eunmi Jeong, Izolda Mileva, Jean-Philip Truman, Chih-li Lin, Kai Wang, Justin Snider, Sally Wen, Lina M. Obeid, Yusuf A. Hannun, Cungui Mao

**Affiliations:** ^1^ Graduate Program in Molecular and Cellular Biology, Stony Brook University, Stony Brook, NY, USA; ^2^ Department of Medicine, Stony Brook University, Stony Brook, NY, USA; ^3^ Stony Brook Cancer Center, Stony Brook, NY, USA; ^4^ Northport Veterans Affairs Medical Center, Northport, NY, USA

**Keywords:** sphingolipids, aging, yeast, sphingoid bases, mitochondria, Gerotarget

## Abstract

Sphingoid bases (SBs) as bioactive sphingolipids, have been implicated in aging in yeast. However, we know neither how SBs are regulated during yeast aging nor how they, in turn, regulate it. Herein, we demonstrate that the yeast alkaline ceramidases (YPC1 and YDC1) and SB kinases (LCB4 and LCB5) cooperate in regulating SBs during the aging process and that SBs shortens chronological life span (CLS) by compromising mitochondrial functions. With a lipidomics approach, we found that SBs were increased in a time-dependent manner during yeast aging. We also demonstrated that among the enzymes known for being responsible for the metabolism of SBs, YPC1 was upregulated whereas LCB4/5 were downregulated in the course of aging. This inverse regulation of YPC1 and LCB4/5 led to the aging-related upregulation of SBs in yeast and a reduction in CLS. With the proteomics-based approach (SILAC), we revealed that increased SBs altered the levels of proteins related to mitochondria. Further mechanistic studies demonstrated that increased SBs inhibited mitochondrial fusion and caused fragmentation, resulting in decreases in mtDNA copy numbers, ATP levels, mitochondrial membrane potentials, and oxygen consumption. Taken together, these results suggest that increased SBs mediate the aging process by impairing mitochondrial structural integrity and functions.

## INTRODUCTION

Aging is an inherently complex process that is manifested by an organism at genetic, molecular, cellular, organ, and system levels. There are three important aspects of the aging process: 1) a continuous decline in biological functions over time, 2) a decreased resistance to multiple forms of stress, and 3) an increased susceptibility to numerous diseases [1]. For example, aging is known to promote various physiological phenomena related to a reduction in the number of cellular tissues as well as body fluid, a decrease of metabolic rate, and loss of biological adaptability [2]. Although a number of studies have been carried out to determine the causes of aging, much remains unclear about the mechanisms that cause aging.

Increasing studies demonstrate that sphingolipids, a group of lipids with SBs as the backbone, may regulate aging process in mammals. It has been shown that ceramides are increased and promote the histochemical senescence marker in aging human fibroblasts [3]. Long-chain hexosylceramides and lactosylceramides were also accumulated during aging in mice and cultured human cells and their accumulation was prevented by the caloric restriction [4]. Furthermore, aberrant accumulation of ceramides has been shown to cause mitochondrial dysfunction by suppressing respiratory chain activity and increasing the generation of ROS in mitochondria isolated from rat heart cells [5]. However, further studies are required to establish the association between the aging process and the sphingolipid metabolism.

The budding yeast, *Saccharomyces cerevisiae,* has been used as a model system to study the aging process because of its short lifespan, the ease of culture, its susceptibility to genetic manipulation, and limited numbers of budding (dividing) [6]. The metabolism of sphingolipids and their roles in regulating biological processes are well conserved between yeast and mammals [7]. Increasing studies suggest that regulation of the metabolism of sphingolipids may affect aging in the yeast system. LAG1 (longevity assurance gene) was the first yeast longevity gene cloned in 1994 [8]. This study found that the transcriptional level of LAG1 is decreased with the replicative age of yeast cells and that deletion of LAG1 extended replicative life span, suggesting that LAG1 may limit yeast longevity. However, the biochemical function of LAG1 was elusive until Brandwagt *et al*. revealed that LAG1 is a ceramide synthase that synthesizes ceramides from fatty acyl-CoAs and LCBs (DHS or PHS) [9]. These observations indicate that ceramides or their derivatives may have a role in regulating aging process in yeast cells. The yeast ISC1 gene was also known to regulate the yeast life span. ISC1 encodes an inositol phosphosphingolipid phospholipase C, an ortholog of mammalian neutral sphingomyelinase-2 that hydrolyzes complex sphingolipids into ceramides [10]. Cells deficient in ISC1 showed a dramatic decline in CLS [11]. Furthermore, it was recently demonstrated that downregulation of LCB1, an essential subunit of the yeast serine palmitoyltransferase (SPT) catalyzing the first step of sphingolipid biosynthesis, can increase the yeast lifespan [12]. Aerts et al. demonstrated that overexpression of Ydc1, which catalyzes the hydrolysis of dihydroceramide into DHS, shortened CLS [13]. These results suggest that sphingolipids may play an important role in regulating yeast aging.

SBs are long-chain aliphatic amines, containing two or three hydroxyl groups. In yeast cells and other low-level eukaryotes, the majority of SBs are DHS and PHS, which are synthesized from serine and palmitoyl-CoA [14] or derived from the hydrolysis of dihydroceramides and phytoceramides, respectively, by the action of the alkaline ceramidases Ydc1p and Ypc1p [15]. Once generated, DHS and PHS are phosphorylated by SPH kinases Lcb4p and Lcb5p to form DHS-1-phosphate (DHS1P) and PHS-1-phosphate (PHS1P), respectively. DHS1P and PHS1P are further cleaved by the SB phosphate lyase Dpl1p to ethanolamine-1-phosphate and aldehydes [16]. Alvarez-Vasquez *et al*. demonstrated that SBs were increased during the diauxic shift, which is considered an early phase of the aging process of the yeast system [17]. It was demonstrated that treatment with PHS promoted the phosphorylation of Sch9p, a target of Tor1p that is known to regulate the yeast lifespan [18]. These results suggest that SBs are upregulated during the aging process and that their upregulation may regulate aging. However, much remains unclear how SBs are upregulated during the aging process and the mechanism by which SBs regulate aging.

Mitochondrial functions and morphology are associated with aging. It is well established that various alterations in mitochondrial structure and functions contribute to aging from yeast to humans [19, 20]. Growing evidence suggests that SBs affect the mitochondrial structural integrity and function. In mammalian cells, SPH was shown to increase the permeability of mitochondria, resulting in the release of cytochrome C from mitochondria in the breast cancer cell line, which in turn activates the intrinsic pathway of apoptosis [21]. In plant cells, treatment with DHS reduced the number of mitochondrial cristae while increasing ROS levels, resulting in apoptosis [22]. It was also demonstrated that a peptide, OSIP108, has a preventive role from an interruption of mitochondrial ultrastructure by ROS and its preventive role was abolished by exogenous DHS [23].

These studies indicated but have not proven that SBs may regulate yeast aging process. In the present study, we show that SBs are persistently accumulated in yeast cells during aging process due to an inverse regulation of Ypc1p and Lcb4p, the enzymes responsible for the generation and degradation of SBs, respectively. More importantly, we further demonstrate that increased SBs shorten CLS by compromising the structural integrity and functions of mitochondria. Our study strongly suggests that SBs and their metabolizing enzymes are important regulators of aging of yeast and possibly other species as well, thus providing novel insights into the molecular mechanisms that control aging.

## RESULTS

### Sphingoid bases and their metabolizing enzymes are regulated in yeast cells during aging process

**Figure 1:**
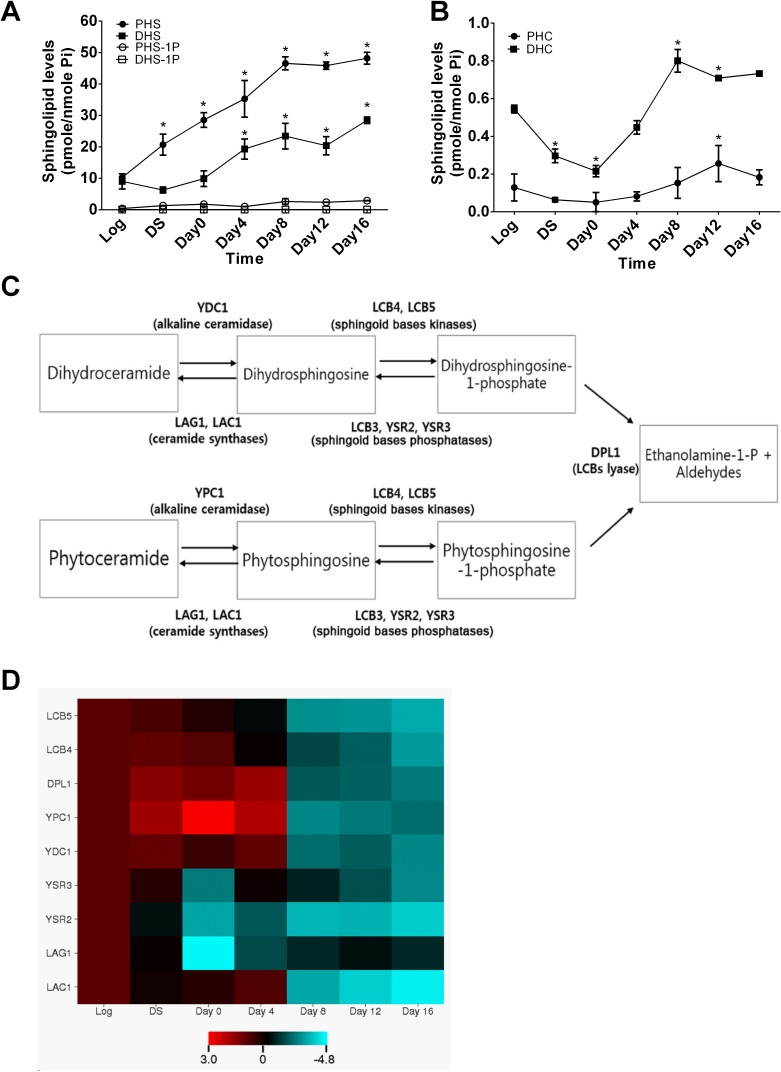
Sphingoid bases and their enzymes are altered in yeast cells during the chronological aging process **A.**and **B.**, aging-related changes in sphingolipid levels in yeast cells. WT JK93d cells were harvested at various time points including the log phase (log), the diauxic shift (DS), the stationary phase (Day 0), and every 4 days post the stationary phase (Day 4, 6, 8, or 16). Cells were subjected to LC-MS/MS analysis for SBs **A.**, PHC **B.**, or DHC **B.** as described in [58]. **C.**, simplified sphingolipid metabolic pathways in yeast cells. **D.**, aging-related changes in mRNA levels of sphingolipid-metabolizing enzymes in yeast cells. JK93d cells grown at different time points were subjected to qPCR analyses for the mRNA levels of sphingolipid-metabolizing enzymes. The heat map was constructed with the log2 ratios of the mRNA levels of each of targeted genes at each post-log phase time point to those at the log phase. Data represent mean ± SD; *n* = 3. *p < 0.05 compared to log phase levels (two-way ANOVA)

Since the metabolism of sphingolipids has been shown to change with age in different species, we wondered if this also occurs in the yeast *Saccharomyces cerevisiae* during the aging process. To this end, we applied a lipidomics approach to measuring the levels of sphingolipids in yeast cells during aging that was induced by nutrient depletion. Wild-type (WT, JK93dα) yeast cells were cultured in the synthetic medium including 2% glucose and harvested at 8 h (corresponding to mid-log phase), 16 h (diauxic shift, DS), 48 h (day 0 in the stationary phase), and 4, 8, 12, and 16 days post the stationary phase before sphingolipid profiling by LC-MS/MS. We found that the levels of PHS were constantly increased from DS up to Day 16 compared to the log phase and that the levels of DHS were increased from Day 0 up to Day 16 (Figure 1A). The levels of phytoceramide (PHC) were decreased from the log phase to Day 0, returned to the log phase levels Day 4, were slightly increased from day 4 to Day 8, and maintained constant from Day 8 to Day 16 (Figure 1B). No significant changes were observed in the levels of dihydroceramides (DHC) during the entire time course (Figure 1B). These results suggest that the levels of SBs (PHS and DHS) are increased in yeast cells markedly and constantly during the entire course of aging.

Following the above finding, we investigated whether enzymes responsible for the metabolism of SBs are also regulated during the aging process. As shown in Figure 1C, the metabolism of SBs involves multiple enzymes, including YPC1, YDC1, LAC1, LAG1, LCB4, LCB5, YSR2/LCB3, and YSR3, so we performed qPCR to determine the mRNA levels of these enzymes in yeast cells collected at different time points as described in Figure 1A and 1B. We found that the mRNA levels of YPC1 were significantly increased in yeast cells at the diauxic shift and the early stationary phase compared to the log phase whereas the mRNA levels of LCB4, LCB5, LAC1, and LAG1 were decreased in yeast cells at the late stationary phase compared to the log phase (Figure 1D). These results suggest that SBs and their producing enzyme YPC1 are upregulated whereas SB-degrading enzymes LCB4/5 are downregulated in yeast cells during the aging process.

### Inverse regulation of alkaline ceramidases and sphingoid base kinases leads to aging-induced elevation of sphingoid bases in yeast cells

Following the above findings, we investigated if increasing YPC1 expression or inhibiting both LCB4 and LCB5 is sufficient to increase the levels of SBs in yeast cells whereas knocking down YPC1 inhibits the aging-related increase in SBs.

**Figure 2:**
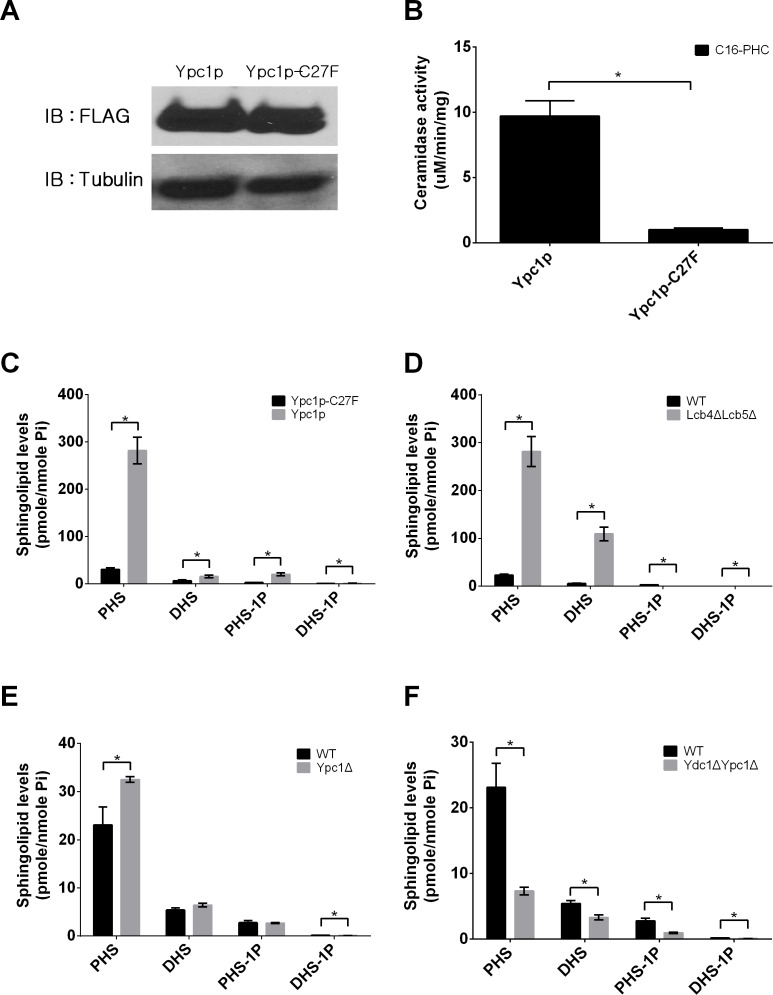
Inverse regulation of YPC1 and LCB4/5 leads to an increase in sphingoid bases in aging yeast cells **A.**, Western blot analyses of Ypc1p and Ypc1p-C27F. YAG6B cells expressing the FLAG-tagged Ypc1p or its mutant, Ypc1p-C27F, were analyzed by Western blot analyses using anti-FLAG antibody as described in Materials and Methods. **B.**, Ypc1p activity assays. Microsomes from cells expressing Ypc1p or Ypc1p-C27F were subjected to alkaline ceramidase activity assays with C_16_-phytoceramide as a substrate as described in Materials and Methods. **C.**, **D.**, **E.** and **F.**, LC-MS/MS analyses of SBs. The levels of SBs were determined by LC-MS/MS in yeast cells overexpressing Ypc1 *vs* Ypc1p-C27F **C.**, WT *vs* Lcb4ΔLcb5Δ **D.**, WT *vs* Ypc1Δ **E.**, or WT *vs* Ydc1ΔYpc1Δ **F.** as described in Materials and Methods. The levels of SBs were normalized to total phosphate. Data represent mean ± SD; *n* = 3. *p < 0.05

First, we determined if YPC1 overexpression could increase endogenous SBs in yeast cells because Ypc1p catalyzes the hydrolysis of phytoceramide to generate PHS. To exclude the possibility that Ypc1p protein *per se* may affect the expression of other LCB metabolic enzymes, a catalytically inactive mutant of this enzyme was constructed as a negative control by site-directed mutagenesis as described in the experimental procedure. We mutated Ypc1p by replacing Cys^27^ with Phe to generate the Cys^27^ > Phe^27^ mutant, named Ypc1p-C27F because it was reported that Cys^27^ is essential for Ypc1p's reverse activity [24]. We generated a yeast stain that overexpresses Ypc1p or Ypc1p-C27F under the control of a Gal1 promoter. These yeast cells were cultured in galactose-containing medium for 8 h to induce gene expression. Protein expression levels and enzymatic activities were examined in microsomes isolated from yeast cells. The expression levels of Ypc1p and Ypc1p-C27F were similar (Figure 2A), and WT Ypc1p but not Ypc1p-C27F exhibited alkaline ceramidase activity (Figure 2B), suggesting that Ypc1p-C27F is a proper negative control for functional studies of the WT Ypc1p. We showed that overexpression of Ypc1p increased the levels of both PHS and DHS in yeast cells compared to overexpression of Ypc1p-C27F (Figure 2C), suggesting that YPC1 upregulation contributes to the increased generation of SBs in yeast cells during the aging process.

Second, we determined if knockout of both LCB4 and LCB5 could also increase the levels of SBs in yeast cells because both enzymes convert SBs to their phosphates [25]. Indeed, with LC-MS/MS, we demonstrated that the levels of SBs were markedly increased in the yeast mutant deficient in both LCB4 and LCB5 compared to WT yeast cells (Figure 2D), suggesting that the downregulation of these enzymes also contributes to an increase in the levels of SBs in yeast cells in response to aging.

Lastly, we determined if YPC1 knockout inhibited the increase in the levels of SBs in yeast cells during aging. Unexpectedly, we found that knocking out YPC1 increased rather decreased the levels of SBs in yeast cells (Figure 2E). Because yeast cells express the other alkaline ceramidase YDC1, which may compensate for the loss of YPC1 for making SBs, we tested if knocking out both YPC1 and YDC1 could lower SBs. Indeed, we found that knocking out both YPC1 and YDC1 markedly inhibited the increase in the levels of SBs in response to aging (Figure 2F). These results suggest that YPC1 and YDC1 have a redundant role in regulating the generation of SBs during the aging process.

### MSN2/4 are required for YPC1 regulation during aging

Following the finding that YPC1 is upregulated during aging, we investigated the mechanism for the YPC1 upregulation. To this end, we tested if MSN2/4 are required for the regulation of YPC1 during the aging process because previous studies indicated that YPC1 is a putative target of MSN2/4, yeast transcriptional factors that regulate expression of various genes implicated in stress response by binding stress response elements (STREs) present on the targeted genes [26, 27]. Interestingly, there are 3 putative STREs within a 100 nucleotide-region in the YPC1 gene (Table S3), indicating that MSN2/4 may regulate YPC1 under stressful conditions.

**Figure 3:**
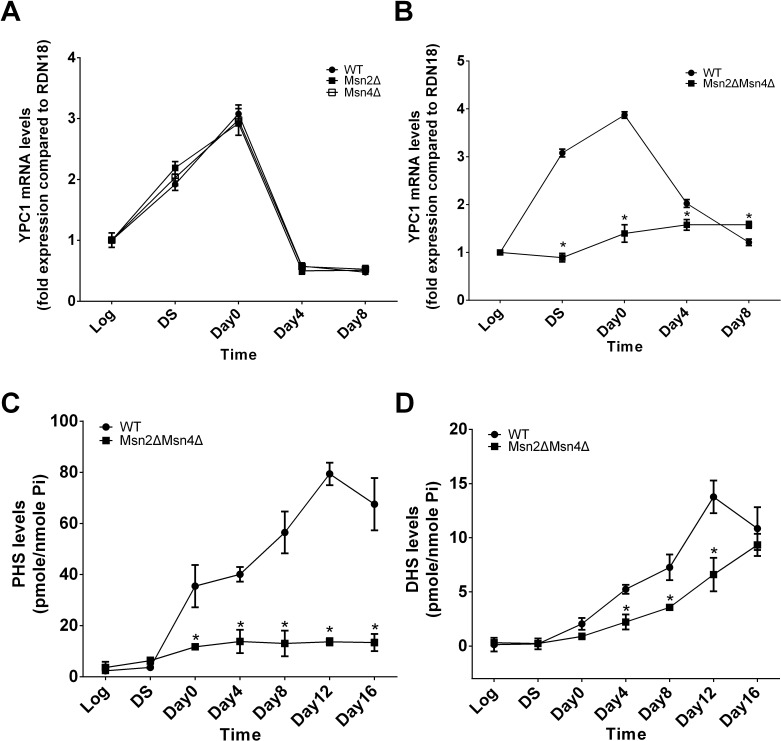
MSN2/4 activation upregulates YPC1 in aging yeast cells **A.** and **B.**, qPCR analyses for YPC1 mRNA levels. WT, Msn2Δ, Msn4Δ, or Msn2ΔMsn4Δ cells were grown in complete SC medium for indicated time durations and YPC1 mRNA levels were determined by qPCR analyses as described in Materials and Methods. Relative YPC1 mRNA levels were analyzed by the 2ΔΔCt method. **C.** and **D.**, LC-MS/MS analyses for SBs. WT and Msn2ΔMsn4Δ cells were harvested at indicated time points and the levels of PHS **C.**, and DHS **D.** were determined by LC-MS/MS analyses as described in Materials and Methods. Data represent mean ± SD; *n* = 3. *p < 0.05

To test this hypothesis, first, we tested if both MSN2 and MSN4 are required to regulate YPC1 mRNA levels during aging. YPC1 mRNA levels were assessed by qPCR in WT, MSN2 single knockout (Msn2Δ, BY4742 background, Table S1), MSN4 single knockout (Msn4Δ, BY4742 background, Table S1), or MSN2 and MSN4 double knockout (Msn2ΔMsn4Δ, W303 background, Table S1) yeast strain at different time points as described earlier. YPC1 mRNA levels were increased with time in WT, Msn2Δ, or Msn4Δ cells but not in Msn2ΔMsn4Δ cells (Figure 3A and 3B), suggesting that MSN2 and MSN4 have a redundant role in regulating YPC1 expression during aging. Second, we tested if MSN2/4 affected SBs levels by regulating YPC1 during the aging process. We analyzed the levels of SBs in WT or Msn2ΔMsn4Δ cells that were grown for different time durations. The results showed that during the aging process, the levels of PHS were increased with time in WT cells but not in Msn2ΔMsn4Δ cells (Figure 3C and 3D). Furthermore, the aging-dependent increase in the levels of DHS was also attenuated in Msn2ΔMsn4Δ cells compared to WT cells. Taken together, these results support the notion that YPC1 is a key enzyme that regulates the aging-associated increase in the levels of SBs in an MSN2/4-dependent manner.

### Upregulation of sphingoid bases reduces yeast chronological life span

Following the finding that SBs and their metabolic enzymes are altered during the aging process, we wondered if these alterations contributed to yeast aging. To this end, we determined if YPC1 overexpression or deletion of both LCB4 and LCB5 accelerated aging by increasing SBs whereas blocking the increase in SBs by inhibiting both YPC1 and YDC1 could delay aging.

**Figure 4:**
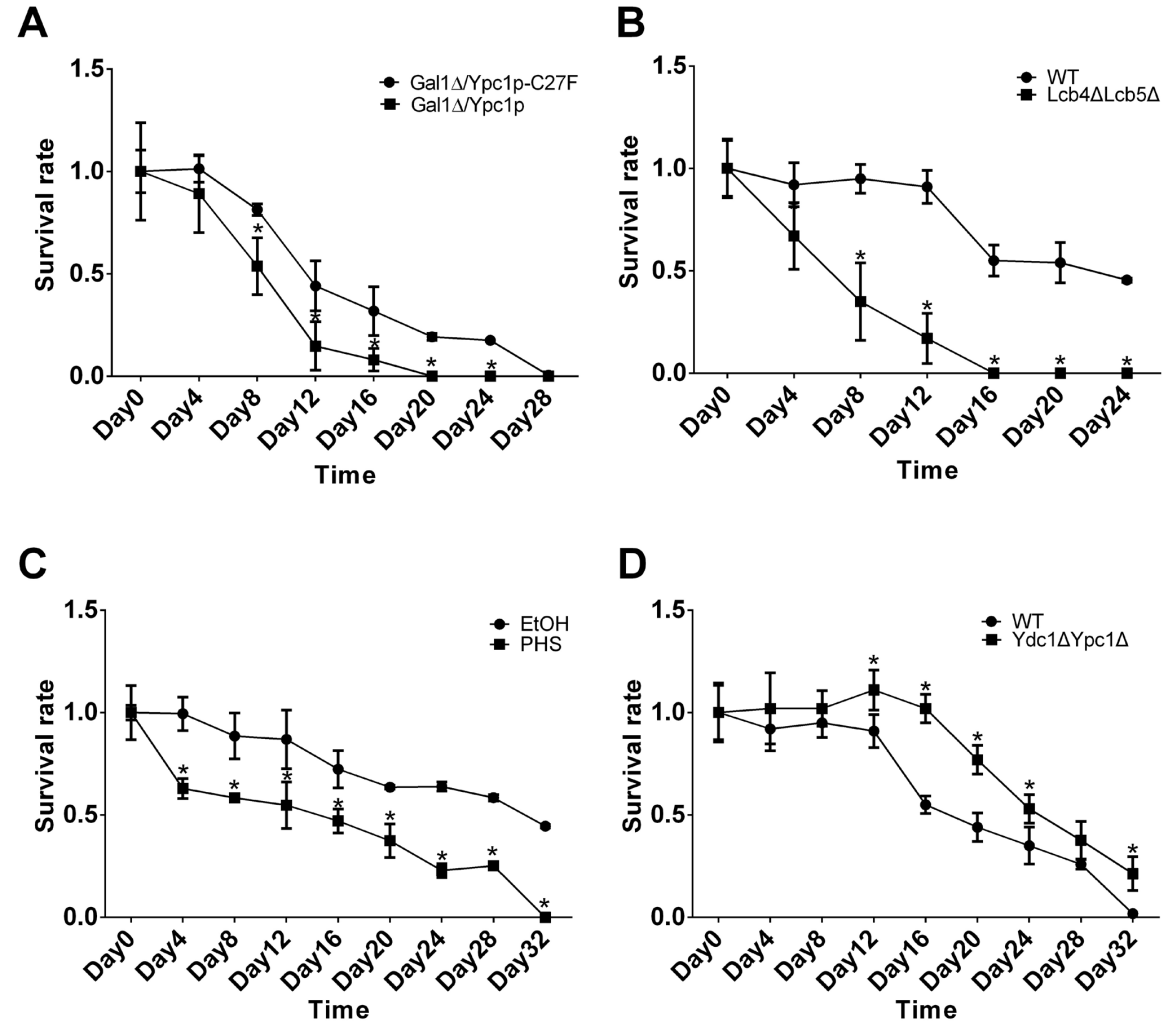
Sphingoid bases decrease CLS **A.-D.** CLS assays of yeast cells. Gal1Δ cells overexpressing either Ypc1p or Ypc1pC27F **A.**, WT *vs*. Lcb4ΔLcb5Δ cells **B.**, WT cells treated with ethanol or PHS (10uM) **C.**, and WT *vs*. Ydc1ΔYpc1Δ **D.** were subjected to CLS assays as described in Materials and Methods. Survival rates of yeast cultures were assayed in triplicate. The data represent the mean ± SD of fold changes in survival rates at indicated time points *vs*. those at the day 0 time point ( *n* = 5), *p < 0.05.

First, we determined if overexpression of YPC1 affected CLS in yeast cells. Galactose is needed to induce YPC1 overexpression, however, its presence in the medium may interfere with CLS assays because galactose can serve as a carbon source to sustain cell growth. For this reason, we constructed a mutant yeast strain (GalΔ) in which galactose cannot be used as a carbon source for growth due to deletion of the Gal1 gene. We transformed Gal1Δ cells with either Ypc1p or Ypc1p-C27F expressing construct to test the effect of YPC1 overexpression on CLS. Western blot analyses demonstrated that the expression of either Ypc1p or Ypc1p-C27F could be induced by galactose to similar levels in GalΔ cells (Figure S1A). We also confirmed that GalΔ cells did not grow in the presence of galactose (Figure S1B). These results suggest that the GalΔ cells expressing Ypc1p and Ypc1p-C27F, respectively, are proper stains for CLS assays. Viability assays showed that GalΔ cells overexpressing WT Ypc1p had a shorter CLS than GalΔ cells overexpressing Ypc1p-C27F, supporting the notion that YPC1 upregulation shortens CLS (Figure 4A).

Second, we determined if knocking out both LCB4 and LCB5 also shortened CLS of yeast cells by increasing SBs. To this end, we performed CLS assays with WT and Lcb4ΔLcb5Δ stains as described earlier. We found that Lcb4ΔLcb5Δ cells had shortened CLS compared to WT cells (Figure 4B), suggesting that similar to YPC1 upregulation, knocking out both LCB4 and LCB5 shortens CLS.

Having demonstrated that overexpression of YPC1 or knocking out LCB4/5 shortens CLS, we wondered that increased SBs are involved in the observed effects. To this end, we determined if PHS, when added exogenously, could shorten CLS. The survival rate of cells treated with PHS (10 μM) or its vehicle, ethanol, was measured. The results showed that treatment with PHS reduced CLS compared to treatment with ethanol (Figure 4C), supporting the notion that the inverse regulation of YPC1 and LCB4/5 contributes to yeast aging by increasing SBs.

To further consolidate the above notion, we determined if the inhibiting aging-related increase in the levels of SBs enhanced CLS. Indeed, we found that Ypc1ΔYdc1Δ cells had a shorter CLS than WT cells (Figure 4D).

In summary, YPC1 overexpression, deletion of both LCB4 and LCB5, or treatment with PHS decreased the yeast life span whereas knockout of both YDC1 and YPC1 increased CLS. These conjoint results strongly suggest that SBs are negative regulators of the yeast CLS.

### Increased sphingoid bases alter levels of proteins related to mitochondrial functions

**Figure 5:**
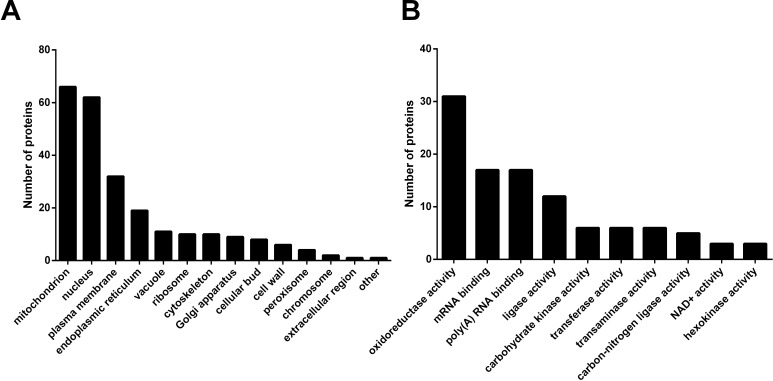
Ypc1p overexpression alters the levels of proteins related to mitochondrial functions Yeast cells overexpressing Ypc1p or Ypc1p-C27F were subjected to SILAC as described in Materials and Methods. **A.**, Bar graph representation of GO annotation results for the cellular components. **B.**, Bar graph representation of GO annotation results for the biochemical functions.

Following the finding that SBs regulate yeast CLS, we wanted to know the underlying mechanism. To this end, we investigated how SBs induced the global changes in proteins through the proteomics-based approach, SILAC as described in Materials and Methods. From the two independent SILAC experiments, we identified 3,570 proteins, among which, 206 proteins showed more than 1.5 folds difference in their expression levels between cells overexpressing Ypc1p and Ypc1p-C27F (Figure S2 and Table S3). Proteins showing different expression levels were categorized regarding their cellular localization and biological functions using the public database (The GO annotation from the *Saccharomyces* Genome Database). The results showed that a cellular component which had the highest cluster frequency was shown to be the mitochondrion (66 out of 206) followed by the nucleus (62 out of 296) and the plasma membrane (32 out of 206), respectively (Figure 5A). Furthermore, the highest frequency in the biological function was shown to be the oxidoreductase activity (Figure 5B), suggesting a possibility that SBs affect mitochondrial functions.

### Sphingoid bases induce fragmentation of the mitochondrial tubular network

Aging is strongly associated with the alterations in the mitochondrial structures [20, 28]. Because SBs increased by the overexpression of YPC1 altered the levels of many mitochondrial and mitochondrion-related proteins, we postulated that increased SBs might alter the mitochondrial morphology and function in aging cells.

**Figure 6:**
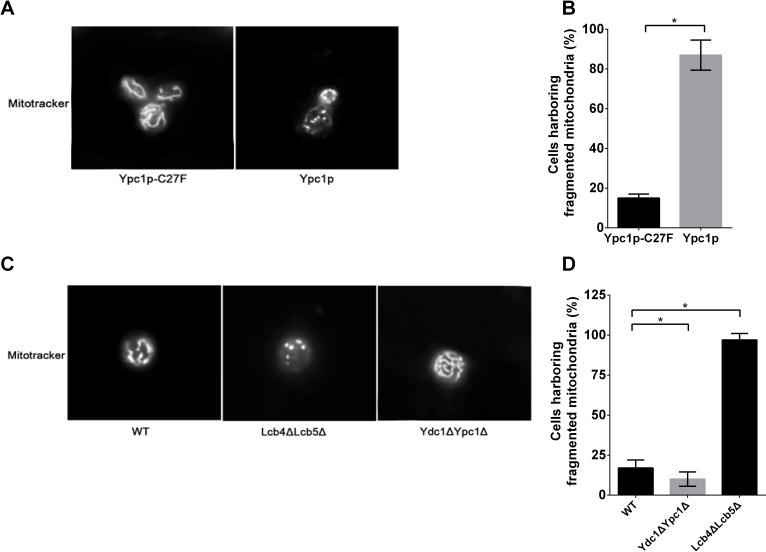
Sphingoid bases alter mitochondrial morphology **A.-D.**, fluorescent microscopic analyses of mitochondrial morphology. Gal1Δ overexpressing either YPC1 or Ypc1p-C27F **A.**, and WT, Ydc1ΔYpc1Δ, and Lcb4ΔLcb5Δ **C.** were labeled with the mitochondrion-specific fluorescent dye, the mitotracker (500 nM), for 30 min before the morphology of mitochondria was observed under a fluorescent microscope as described in Materials and Methods. The numbers of cells harboring fragmented mitochondria were counted under a fluorescence microscope and expressed as a percentage of the total numbers of cells overexpressing Ypc1p or Ypc1p-C27F **B.**, WT, Lcb4ΔLcb5Δ, or Ydc1ΔYpc1Δ cells **D.**. Data represent mean ± SD; 10 random fields with 100x magnification. *p < 0.05

To test this possibility, we first observed the mitochondria morphology by fluorescent microscopy using mitotracker labeling. Cells overexpressing Ypc1p or Ypc1p-C27F were collected 12 h after gene expression induction (late log phase) and stained with the mitotracker before being observed for mitochondrial morphology under a fluorescent microscope. The results showed that cells overexpressing Ypc1p-C27F had an intact mitochondrial tubular network whereas cells overexpressing Ypc1p had a fragmented mitochondrial network (Figure 6A and 6B), suggesting that increased SBs disrupt the integrity of the mitochondrial network.

To further verify the effect of SBs on the mitochondrial morphology, the morphology of the mitochondrial network was compared between Lcb4ΔLcb5Δ cells and WT cells in the late log phase in SDC medium. The results showed that the mitochondrial network was severely fragmented in Lcb4ΔLcb5Δ cells whereas this network was intact in WT cells (Figure 6C and 6D), confirming increased SBs indeed disrupt the integrity of mitochondria in cells.

To further confirm this notion, we investigated if inhibiting the generation of SBs by knocking out both YPC1 and YDC1 can attenuate mitochondrial fragmentation in aging cells. To this end, the integrity of the mitochondrial network was assessed in Ydc1ΔYpc1Δ and WT cells during the aging process. Ydc1ΔYpc1Δ and WT cells were incubated in SDC medium until the late log phase before they were stained with the mitotracker. Fluorescent microscopy revealed that the mitochondrial network was fragmented in WT cells, and this was significantly attenuated in Ydc1ΔYpc1Δ cells (Figure 6C and 6D), confirming the notion that the aging-related increase in SBs induces the fragmentation of the mitochondrial tubular network in yeast cells.

### Sphingoid bases compromise mitochondrial functions

The functions of mitochondria are highly dependent on their structural integrity. Having shown that SBs alter the mitochondrial morphology, we investigated if increased SBs altered the mitochondrial functions, such as mitochondrial respiration, oxygen consumption rates, ATP production, and mitochondrial Δψm.

**Figure 7:**
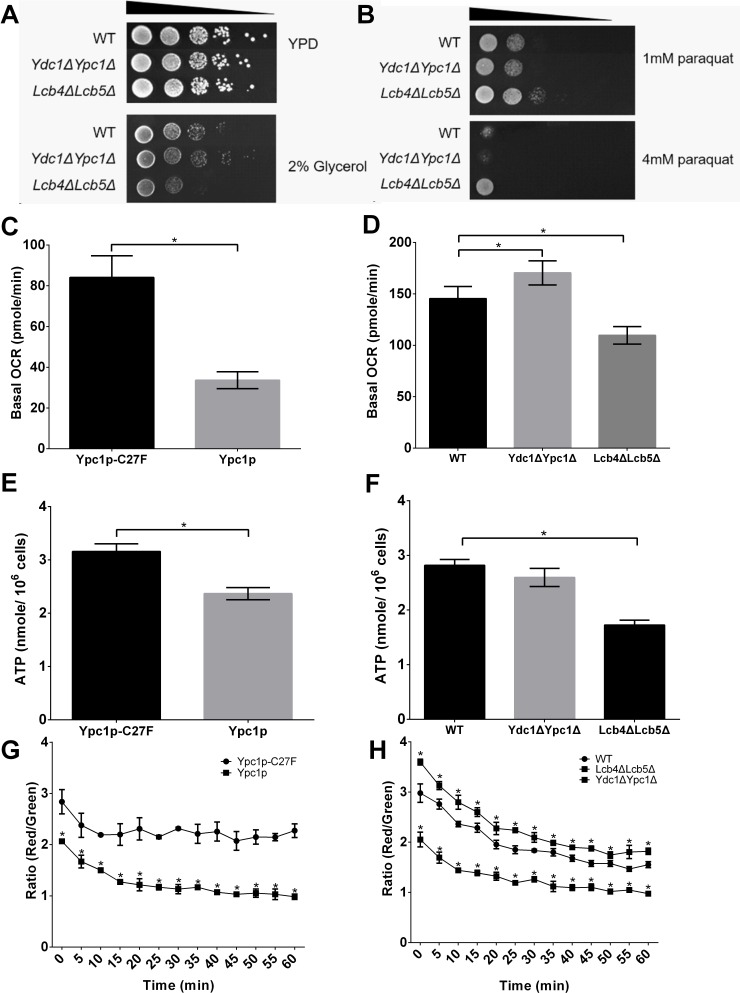
Sphingoid bases impair mitochondrial functions **A.**, Utilization of nonfermentable carbon sources of mutant yeast cells. Serial dilutions of mid-log phase cultures containing 10^6^-10^2^ cells (WT, Lcb4ΔLcb5Δ, and Ydc1ΔYpc1Δ) were spotted onto YPD and YEP plates containing 2% (wt/vol) glycerol. **B.**, Sensitivity to oxidative inducing-agent (paraquat) of yeast cells. Serial dilutions of mid-log phase cultures containing 10^6^-10^2^ cells (WT, Lcb4ΔLcb5Δ, and Ydc1ΔYpc1Δ) were spotted onto YPD plates containing either 1 mM or 4 mM paraquat. **C.** and **D.**, Basal oxygen consumption. **E.** and **F.**, intracellular ATP assays. **G.** and **H.**, mitochondrial membrane potential (Δψm) assays. Yeast cells overexpressing Ypc1p or Ypc1p-C27F (C, E, and G) were grown overnight (O/N) in SC medium and WT, Ydc1ΔYpc1Δ, or Lcb4ΔLcb5Δ in complete SC medium (D, F, and H) before yeast cultures were subjected to OCR assays (C and D), intracellular ATP assays (E and F), or Δψm assays (G and H) as described in Materials and Methods. Data represent mean ± SD; *n* = 9 for the OCR assays, *n* = 6 for the ATP assay, and *n* = 12 for the Δψm assay. *p < 0.05

To establish whether SBs affected mitochondrial respiration, the growth on an oxidative carbon source, glycerol, were determined using WT, Ydc1ΔYpc1Δ, and Lcb4ΔLcb5Δ cells. Compared to WT, the growth of Ydc1ΔYpc1Δ cells was elevated, whereas Lcb4ΔLcb5Δ showed the high sensitivity to oxidative carbon source (Figure 7A), suggesting that SBs induce mitochondrial respiration deficiency. To further verify the effect of SBs on mitochondria respiration, the oxygen consumption rates (OCR) were assessed in different yeast stains by XF96 mitochondrial flux analyzer. We found that cells overexpressing Ypc1p had a lower oxygen consumption rate than cells overexpressing Ypc1p-C27F (Figure 7C). Consistently, compared with WT cells, Lcb4ΔLcb5Δ cells showed a significantly lower OCR (Figure 7D). These results strongly suggest that increased SBs indeed inhibit oxygen consumption, a key mitochondrial function.

Because the respiration is proportional to the ATP generation [29], we then determined if increased SBs lowered ATP levels in yeast cells. We measured the intracellular ATP levels in different yeast strains. First, we determined if Ypc1p overexpression reduced ATP levels in yeast cells in the log phase. Yeast cells expressing Ypc1p or Ypc1p-C27F were harvested at the late log phase before they were subjected to ATP measurements. As shown in Figure 7E, cells overexpressing Ypc1p had lower ATP levels than cells overexpressing Ypc1p-C27F. We then determined if knocking out LCB4 and LCB5 had a similar effect on ATP level to Ypc1p overexpression. WT or Lcb4ΔLcb5Δ cells were grown to the late log phase before they were subjected to ATP measurements. As shown in Figure 7F, Lcb4ΔLcb5Δ cells had lower ATP levels than WT cells, confirming that increased SBs inhibit ATP production.

Because the Δψm is regarded as the main driving force for ATP production [30], we decided to measure Δψm by JC-1 staining to verify the effect of increased SBs on Δψm as well. Mitochondria were isolated from different yeast strains and assayed for Δψm. We found that cells overexpressing Ypc1p showed lower Δψm than cells overexpressing Ypc1p-C27F (Figure 7G). Consistently, Lcb4ΔLcb5Δ cells showed a significantly lower Δψm than WT cells (Figure 7H). In contrast, Ydc1ΔYpc1Δ cells had higher Δψm than WT cells (Figure 7H).

It is also known that yeast cells defective in mitochondrial respiration are resistant to paraquat because paraquat-induced superoxide production required Δψm that was essential for paraquat uptake into mitochondria [31, 32]. In the spot assay, Lcb4ΔLcb5Δ showed the highest resistance to the paraquat among other cells including WT and Ydc1ΔYpc1Δ. Moreover, the growth of Ydc1ΔYpc1Δ cells in the presence of 4 mM paraquat was almost abolished (Figure 7B), verifying that the relatively intrinsic Δψm was increased in Ydc1ΔYpc1Δ cells. Taken together, these results suggest that increased SBs indeed compromise mitochondrial functions by decreasing Δψm, intracellular ATP levels, and basal OCR.

### Sphingoid base levels are increased in mitochondria during yeast aging

**Figure 8:**
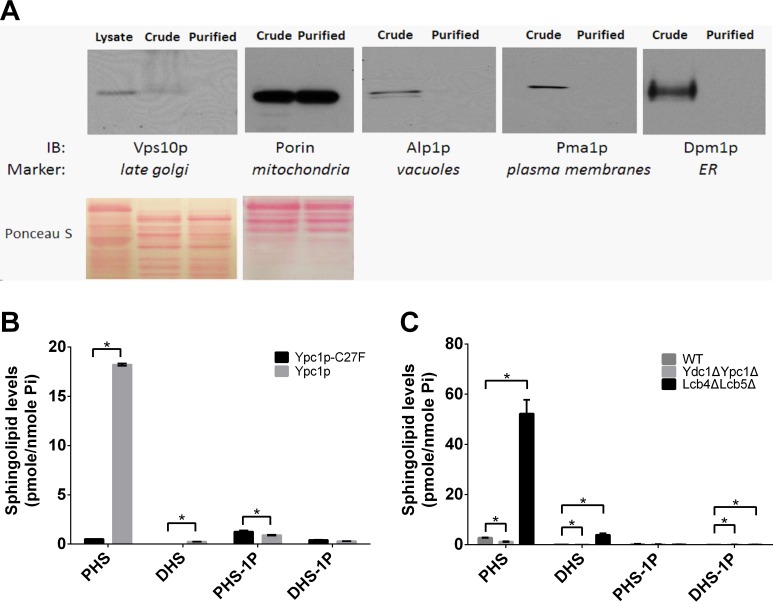
Sphingoid base levels are increased in mitochondria in aging yeast cells **A.**, Isolation of mitochondria. Mitochondria were isolated from yeast cells and their purity was confirmed by Western blot analyses with antibodies against different organelle-specific proteins as described in Materials and Methods. **B.** and **C.**, LC-MS/MS analyses of mitochondrial SBs. LC-MS/MS was performed to determine the levels of SBs in mitochondria isolated from cells overexpressing Ypc1p or Ypc1p-C27F **B.**, WT, Lcb4ΔLcb5Δ, or Ydc1ΔYpc1Δ cells **C.** as described in Materials and Methods. Data represent mean ± SD; *n* = 3. *p < 0.05

Because increased SBs impair both mitochondrial structure and functions, we wondered if SBs are increased in mitochondria in aging cells. We measured the levels of SBs from mitochondria purified from different yeast strains by the sucrose gradient ultracentrifugation as described [33]. Immunoblotting was performed to verify the purity of the purified mitochondria using antibodies against porin, Alp1p, Pma1p, Dpm1p, or Vps10p, which are markers for various organelles including mitochondria, vacuoles, plasma membranes, ER or late Golgi, respectively. We found that the mitochondrial marker porin but not the makers for the other organelles was detectable in the mitochondrial preparations, suggesting that the mitochondria were highly purified (Figure 8A). With LC-MS/MS, we found that the levels of SBs were increased in mitochondria from cells overexpressing Ypc1p compared to cells overexpressing Ypc1p-C27F (Figure 8B). Consistently, compared with WT cells, Lcb4ΔLcb5Δ cells showed significantly higher SB levels whereas Ydc1ΔYpc1Δ cells showed lower SBs levels in the mitochondria (Figure 8C). These results suggest that SBs produced in either the ER or Golgi complex by the alkaline ceramidases are transported to mitochondria and act locally to impair mitochondrial morphology and functions.

### Sphingoid bases inhibit mitochondrial fusion and decrease mtDNA copy numbers

**Figure 9:**
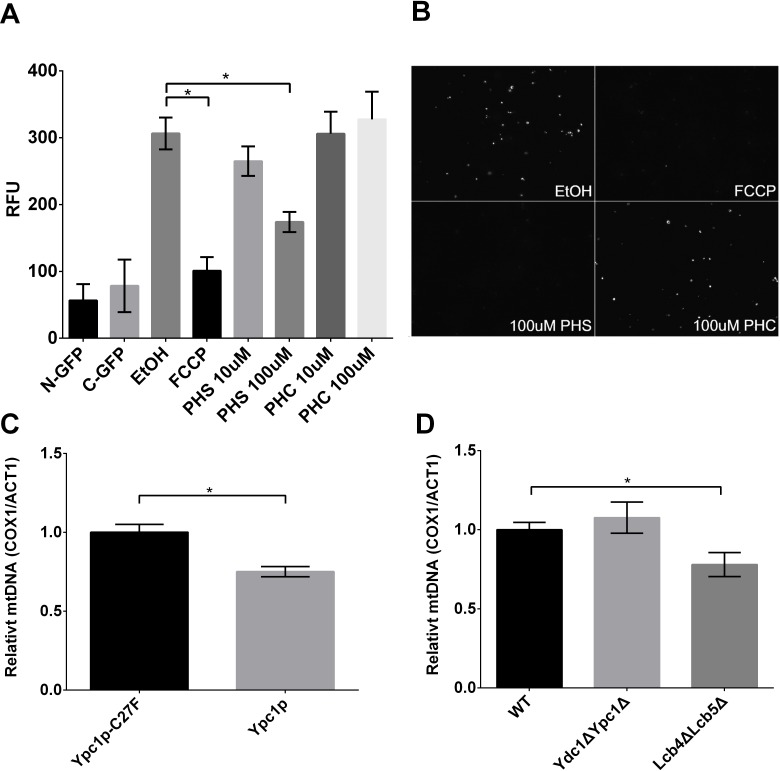
Sphingoid bases inhibit mitochondrial fusion and decreases mtDNA copy number **A.**, *in vitro* mitochondrial fusion assay. Isolated mitochondria were treated with different concentrations of PHS, and mitochondrial fusion was assayed by GFP florescence measurements as described in Materials and Methods. Data represent mean ± SD; *n* = 6. *p < 0.05. **B.**, representative GFP fluorescent images of mitochondrial fusion reactions. C and D, mtDNA quantification in cells overexpressing Ypc1p or Ypc1p-C27F **C.**, WT, Lcb4ΔLcb5Δ, or Ydc1ΔYpc1Δ cells **D.**. Relative mtDNA copy numbers were analyzed by a 2ΔΔCt method using RT-qPCR data. Data represent mean of ± SD; *n* = 3. *p< 0.05.

Because altered SBs are known to affect membrane structure and permeability [34], we investigated whether an increase in the mitochondrial SBs affected mitochondrial fusion using *in vitro* mitochondrial fusion assays. Mitochondria were isolated from yeast cells expressing N-GFP and C-GFP respectively and combined to initiate mitochondrial fusion in the presence of various concentrations of PHS. We found that PHS inhibited mitochondrial fusion in a concentration-dependent manner (Fig 9A and 9B), suggesting that increased SBs indeed inhibit mitochondrial fusion. To verify the inhibition of mitochondrial fusion is a specific effect of PHS, we also treated mitochondria with C_8:0_-phytoceramide (C_8:0_-PHC). Interestingly, there was no significant inhibition of mitochondrial fusion upon the C_8:0_-PHC treatment (Figure 9A and 9B). This result indicates that mitochondrial fusion is specifically induced by PHS.

Because mitochondrial fusion is strongly associated with mtDNA maintenance [35, 36], we then sought to determine if inhibition of mitochondrial fusion caused by SBs leads to the decreased number of mitochondria using a qPCR. It was observed that mtDNA copy number was reduced in *YPC1* overexpressing cells by 25% compared with control cells (Figure 9C). Lcb4ΔLcb5Δ also showed a decrease in mtDNA copy number compared with WT cells (Figure 9D). These results suggest that increased SBs due to the inverse regulation of YPC1 and LCB4/5 reduce the mtDNA copy number likely by inhibiting mitochondrial fusion.

## DISCUSSION

Sphingolipid metabolites, such as ceramides and SBs, have shown to act as bioactive lipids to accelerate aging. However, much remains unclear about how these sphingolipids and their metabolizing enzymes are regulated during aging processes. In this study, we for the first time demonstrate that SBs are markedly increased in yeast cells during the course of nutrient deprivation, a stress that induces aging, due to an inverse regulation of their producing enzymes (the alkaline ceramidases, YPC1 and YDC1) and their degrading enzymes (the SB kinases, LCB4 and LCB5). More importantly, this study reveals that increased SBs shorten yeast CLS by impairing both the structural integrity and functions of mitochondria, thus, playing a key role in mediating yeast aging.

Sphingolipids have been known to be altered in different organisms in response to the aging. Mouton *et al*. found that ceramides are increased in human fibroblasts during aging [3]. Furthermore, it has been shown that the levels of long-chain ceramides are increased in mice during a normal aging [37]. Our recent studies demonstrated that SPH levels are also increased in the brain in middle-aged mice compared to young adult mice [38]. In this study, we demonstrated that SBs were increased during the aging process in JK-93d yeast cells (Figure 1) and several other yeast strains (data not shown). However, we found that the levels of phytoceramides, the yeast ceramides, were transiently decreased in yeast cells from the log phase to the stationary phase and were only slightly increased in the late phase of the aging process. These results suggest that aging process-associated increase of SBs is a highly conserved phenomenon across various species.

SBs are intermediates in the metabolic network of sphingolipids so their levels can be controlled by different sphingolipid-metabolizing enzymes. In this study, we for the first time demonstrated that upregulation of the alkaline ceramidases YPC1 and YDC1 is a major cause for the aging-associated increase in the levels of SBs in yeast cells. First, we showed that the mRNA levels of YPC1 are increased during the early phase of aging. Second, knocking out both YPC1 and YDC1 but no single ceramidase totally blocked the aging-associated increase in the levels of SBs in yeast cells, suggesting that the hydrolysis of yeast ceramides by the action of both YPC1 and YDC1 is the major source of SBs that are increased in response to aging. Interestingly, Zhao et al. demonstrated that different types of ceramidases are also upregulated in rat aging brains compared to young brains. [39]. Our recent study found that the alkaline ceramidase ACER3, a human homologue of the yeast alkaline ceramidases, is also significantly upregulated in aging mouse brains compared to young brains [38]. These results suggest that it is a general phenomenon for SB-producing ceramidases to increase in different species during aging. In addition to ceramidases, SB kinases LCB4/5 are important in regulating the levels of SBs by controlling SB phosphorylation. In this study, we for the first time demonstrated that downregulation of LCB4/LCB5 also plays an important role in increasing the levels of SBs during the late phase of aging. Although Lester et al. already showed that LCB4 deletion induced the increase in SBs during the diauxic shift [40], it was required to check its role in SB regulation for the longer period of time in order to study aging. First, we showed that both LCB4 and LCB5 mRNA levels were decreased in yeast cells during aging process, especially in the late phase. Second, knocking out both LCB4 and LCB5 is sufficient to increase SBs in yeast cells even in the log phase. This explains why SBs continue to be increased in yeast cells in the late phase of aging although SB-producing enzymes YPC1 and YDC1 are not upregulated. Taken together, these results strongly suggest that an inverse regulation of the ceramidases and SB kinases is responsible for the aging –associated increase in the levels of SBs in yeast cells.

Previous studies demonstrate that the transcription factors MSN2/4 play a key role in regulating the expression of genes implicated in stress responses of yeast cells. These stress-responsive transcriptional activators, MSN2/4, are known to bind to STRE [41]. There are several putative STREs found in the promoter region of YPC1. A previous study showed that a loss of one of such STREs leads to 36% reduction in YPC1 gene expression [27]. Interestingly, our study showed no difference in YPC1 mRNA levels between WT and a single deletion strain of either MSN2 or MSN4 but, the deletion of both MSN2 and MSN4 totally blocked the upregulation of YPC1 in yeast cells in response to prolonged culture in nutrient-deficient medium, indicating that MSN2/4 have a redundant role in regulating the expression of YPC1 in response to nutrient depletion. Although MSN2/4 activity was shown to protect yeast cells from the detrimental effects of extreme stress [26, 42] and to be required to extend the life span [43, 44], our result showed a small but significant increase in CLS in Msn2/4Δ. There are also a couple of previous studies showing no or little difference in CLS between WT and Msn2/4Δ [43, 45]. Moreover, it was reported that deletion of SIR2 which is subsequently activated by a target of MSN2/4, PNC1, can extend chronological lifespan in several strains [46]. Because MSN2/4 regulate expressions of various genes under different stresses [47] and the difference in yeast backgrounds may affect the outcome of an experiment, our results might be contradictory to previous studies where deletion of MSN2/4 activities were required to extend yeast CLS. Taken together, YPC1 and its lipid products SBs are upregulated by MSN2/MSN4 in yeast cells in response to nutrient depletion and possibly other forms of stress.

Studies from different groups demonstrate that knocking out different sphingolipid-metabolizing enzymes affects yeast replicative or chronological lifespan [4, 11-13, 34, 48-51], suggesting that sphingolipids may regulate yeast aging. However, what exact sphingolipids regulate yeast aging remains largely unclear although previous studies implied but have not proven that SBs may regulate yeast aging [12]. In this study, we provided strong evidence that SBs produced by the action of YPC1 and YDC1 regulate yeast CLS. First, we demonstrated that SBs but not yeast ceramides were increased during yeast chronological aging. Second, we showed that knocking out both YPC1 and YDC1 inhibited the chronological aging-associated increase in the levels of SBs and prolonged CLS. Third, enforced increases in the levels of SBs in yeast cells due to either YPC1 overexpression or deletion of both LCB4/LCB5 shortened yeast CLS. Finally, treatment with exogenous PHS shortened yeast CLS. Because we previously showed that the mammalian SB, SPH, is also increased the mouse aging brain, we speculated that this age-associated increase in SPH may contribute to physiological or pathological aging of mammals. This view appears to be supported by recent studies showing that knocking out the S1P lyase gene (SGPL1) markedly increased the levels of free SBs (SPH and DHS) in addition to LCB phosphates (S1P and DHS1P) [52], thus shortening the lifespan of mice [53, 54].

Although SBs are known to inhibit cell growth and induce apoptosis [55], the underlying mechanism by which SBs regulate yeast aging remains unclear. Using a proteomic approach, SILAC, we found that the expression of various proteins important for maintaining the mitochondrial morphology and functions are altered upon YPC1 overexpression (Fig 5). This suggests to us that SBs may alter the mitochondrial structure and functions. Indeed, we provided strong evidence that SBs produced by the action of YPC1 and YDC1 are transported to the mitochondria and inhibit mitochondrial fusion and mitochondrial functions. First, we showed that YPC1 overexpression increased the levels of PHS and DHS in the yeast mitochondria. Second, either overexpression of YPC1 or deletion of LCB4/5 induced fragmentation of the mitochondria. Consistently, Aerts et al. showed that overexpression of YDC1 (which may increase the levels of DHS in yeast cells by catalyzing the hydrolysis of dihydroceramides) induced mitochondrial fragmentation [13]. Third, knocking out both YPC1 and YDC1 inhibited fragmentation of the mitochondria in aging yeast cells. Fourth, our *in vitro* studies showed that PHS inhibited the mitochondrial fusion in the test tube. These results strongly suggest that SBs may directly target on the mitochondrial membrane and alter the structural integrity of the mitochondria. Because the proper structure of the mitochondria is essential for mitochondrial functions, it is conceivable that SBs may also compromise the mitochondrial functions. Indeed, we showed that that either YPC1 overexpression or LCB4/5 deletion decreased the intracellular levels of ATP and inhibited mitochondrial respiration. Because numerous studies demonstrated that declined mitochondrial functions can promote aging process [56, 57], we postulated that increased SBs due to the inverse regulation of the ceramidases and SB kinases shortens yeast CLS at least in part by compromising both the structural integrity and functions of the mitochondria.

In conclusion, the data presented in this study strongly suggests that increased SBs due to the inverse regulation of their producing enzymes YPC1 and their degrading enzymes LCB4 and LCB5 shortens yeast CLS by compromising the mitochondrial fusion and functions. This important finding may facilitate our understanding of the role and mechanism of the action of sphingolipids in physiological and pathological aging of humans and other mammals.

## MATERIALS AND METHODS

### Reagents

A mouse monoclonal anti-FLAG antibody, horseradish-peroxidase couple anti-rabbit and anti-mouse antibodies were purchased from Santa Cruz Biotech, TX. Anti-tubulin, anti-Vps10p and anti-PMA1 antibodies were obtained from Abcam, MA. Anti-ALP, anti-porin and anti-dpm1p antibodies were purchased from Thermo Fisher Scientific, MA. D-*erythro*-sphingosine, D-*ribo*-phytosphingosine and C_16:0_-phytoceramide were obtained from Avanti Polar Lipids, AL. L-Lysine (^13^C^6^) and L- Arginine (^13^C^6^) were obtained from Cambridge Isotope laboratories Inc, MA.

### Yeast strains, media and growth conditions

Yeast strains used in this study are presented in Table S1. The wild-type *Saccharomyces cerevisiae* strains JK93d and YAG6B were grown in YPD medium (1% yeast extract, 2% peptone, and 2% dextrose), and transformed strains a uracil dropout medium SC-ura (CLONTECH, CA). Yeast cells were cultured at 30°C under rotational shaking at 200 rpm. The gene overexpression strains in this study constructed as follows. To overexpress Ypc1p in yeast cells under the control of the promoter Gal1, the open reading frame (ORF) of the YPC1 gene was cloned into the vector pYES2 to yield the construct pYES2::YPC1 as described in [15] and the resulting expression construct pYES2::YPC1 was sequenced to ensure that no errors were introduced into the YPC1 ORF by PCR before being transformed into yeast cells by the lithium acetate method [15]. The strain containing pYES2::YPC1 was grown and maintained in SC-ura medium with 2% glucose. Expression of Ypc1p was induced in SC-ura medium with 2% galactose. To exclude the possibility that Ypc1p *per se* may induce biological effects independently of its lipid mediator, a catalytically inactive mutant of this enzyme was constructed as a negative control. Ypc1p was mutated by replacing Cys^27^ with Phe to generate the Cys^27^ > Phe^27^ mutant [24], named Ypc1p-C27F, by a QuikChange II XL Site-Directed Mutagenesis Kit (Agilent Technology, CT) following the manufacturer's manual. The ORF of Ypc1p-C27F was cloned into a pYES2 vector, and the resulting expression construct, pYES2::YPC1^C27F^, was transformed into yeast cells as described previously.

### CLS assays

CLS assays were performed in cells grown in liquid synthetic complete medium containing 2% glucose (SDC) supplemented with standard amounts of amino acids. Briefly, yeast strains from frozen stock (−80°C) were streaked onto either YPD agar plates or SD-ura plates containing 2% glucose and incubated at 30°C. The following day, cells were inoculated into 5 ml of either SDC medium or SD-ura and grown overnight. The overnight culture was inoculated into 50 ml of either SDC medium or SD-ura medium in a 250-ml flask to an optical density at 600 nm (OD600) of 0.1, and the flask was shaken at 250 rpm at 30°C. Under these culture conditions, yeast cultures reached a maximum cell density at 48 hours, so Day 3 after inoculation was considered as Day 0 of CLS. Only for the transformed cells, galactose was added into the medium at Day 0 to induce gene overexpression. Subsequently, cellular viability was determined on different days by colony formation unit (CFU) assays. Briefly, cell number was estimated by optical density (OD) for each population and serial dilutions of different cultures were plated onto 3 YPD plates. Plates were incubated at 30°C for 48 hours before CFU were counted.

### Mitochondria isolation

Mitochondria were isolated from yeast cells by the yeast mitochondria isolation kit (BioVision, CA) according to the manufacturer's instructions. Although the resulting samples are enriched in mitochondria, they may contain other organelles such as the endoplasmic reticulum, Golgi complex, and vacuoles. To obtain pure mitochondria, this crude mitochondrial fraction was subjected to further fractionation as described [33]. Briefly, a sucrose gradient was constructed by multiple layers extending from 60% sucrose to 15% sucrose in EM buffer (10 mM MOPS/KOH (pH 7.2), 1 mM EDTA) in a Beckman Ultra-Clear centrifuge tube (Beckman Coulter Life Sciences). Three ml of the crude mitochondrial fraction in SEM buffer (10 mM MOPS/KOH, pH 7.2, 250 mM sucrose, and 1 mM EDTA) was placed on the top of 15% (w/v) sucrose and centrifuged in a Beckman SW44 Ti swinging-bucket rotor for 1 h at 134,000g at 4°C. The intact mitochondria were obtained from a brown band at the 60%/32% sucrose interface, and purified mitochondria were used for sphingolipid analyses, mitochondrial membrane potential assays, and mitochondrial fusion assays.

### Sphingolipid analyses

Yeast cells were harvested at various time points including the log phase, the diauxic shift, and every 4 days in the stationary phase. The cells were washed and suspended in a lipid extraction solvent consisting of ethanol, diethyl ether, pyridine, ammonium hydroxide, and water (50:10:2:25:15 by volume) in a screw-cap vial containing 0.5 ml of glass beads (Sigma, MO) before being homogenized for 10 min by vortexing at 4°C at the maximum speed. For mitochondrial lipid analyses, mitochondria were extracted with the solvent system consisting of 70% isopropanol: ethyl acetate: pyridine: 25% ammonia (60:20:2:0.5 by volume). The lipid extracts were dried under a stream of N_2_ gas and reconstituted in 150 μl of methanol before sphingolipids were determined by LC-MS/MS performed on a TSQ Quantum Ultra quadruple mass spectrometer (Thermo Finnigan, NJ) according to the method described in [58].

### Quantitative mRNA levels of sphingolipid metabolism enzymes

Total RNA was extracted from yeast cells harvested at different time points as described in [59]. Oligonucleotide primers for RT-qPCR were described in Table S2. One microgram of total RNA was reverse-transcribed into cDNA in a 20 μl reaction mixture using the SuperScript® III Reverse Transcriptase (Life Technology, CA). The reaction mix (25 μl final volume) consisted of 12.5 μl of iQ SYBR Green Supermix (Bio-Rad, CA), 1.25 μl of each primer (0.5 μM final concentration), and 10 μl of a 1/10 dilution of the cDNA preparation. The thermocycling program consisted of one hold at 95°C for 3 min, followed by 40 cycles of 10 s at 95°C and 45 s at 60°C. Relative gene expression levels were determined by the 2ΔΔCt method [60].

### Ceramidase activity assay

Ceramidase activity was assayed as described in [38] with slight modifications. C_16_-phytoceramide (2.5 nmoles) in ethanol was dried under a stream of nitrogen gas, resuspended in 40 μl of the assay buffer A (25 mM Tris-HCl, pH 8.0, 5 mM CaCl, and 0.5% Triton X-100), boiled for 10 s, and chilled on ice, and sonicated in a water bath ultrasonicator for 2 min as described [61]. The substrate was incubated with 40 μl (approximately 400 μg of proteins) of microsomes at 37°C for 60 min, and the reaction was terminated by adding 400 μl of methanol. The reaction mixture was dried and dissolved in 30 μl of chloroform:methanol (2:1, v/v) before PHS was quantified by LC-MS/MS as described in [58].

### Stable isotope labeling by amino acids in cell culture (SILAC)

The yeast strain YAG6B, which is auxotrophic to both lysine and arginine [62], was transformed with pYES2::YPC1 or pYES2::YPC1^C27F^. Yeast cells overexpressing YPC1 and YPC1^C27F^ were grown in a medium containing 100 mg/L arginine and lysine or 100 mg/L [^13^C^6^] arginine and [^13^C^6^] lysine for more than 10 generations to ensure that the most of amino acids in cells were replaced with ones from medium. Proteins were extracted from yeast cells and were separated on a 10% sodium dodecyl sulfate polyacrylamide gel electrophoresis (SDS-PAGE). The gel was stained with Coomassie Blue and excised into 5−10 small slices. Each gel slice was further cut into 1 mm^3^ cubes, in which proteins were digested by trypsin into peptides as described in [63]. Peptides resulting from trypsin digestion were analyzed by liquid chromatography mass spectrometry with very high mass accuracy and sequencing speed. Tryptic peptides were analyzed on a LTQ-Orbitrap XL mass spectrometer (Thermo Fisher Scientific, MA). The mass spectrometer was programmed to operate in data-dependent mode, such that the top 10 most abundant precursors in each MS scan were subjected to CAD (electron multiplier detection, collision energy = 35%, isolation width = 2.8 Da, threshold = 20000). Dynamic exclusion was enabled with a repeat count of 1 and exclusion duration of 15 seconds. Raw data files were loaded directly into PEAKS 7 software (Bioinformatics Solutions Inc., ON, Canada) where the data were refined and subjected to de novo sequencing and database searching. Search parameters included trypsin specificity, up to 2 missed cleavages, fixed carbamidomethylation (C, +57 Da). Oxidation (M, +16 Da) was further specified as variable modifications. The tolerance values used were 10 ppm for parent ions and 0.6 Da for fragment ions. Since proteins were isotopically labeled, variable modification for lysine (+4.03 and +8.01 for light and heavy label, respectively) and arginine (+6.02 and 10.02 for light and heavy label, respectively) were also considered. The resulting peptide sequences were searched against a forward-reversed UniProt database consisted of *Saccharomyces cerevisiae* sequence (downloaded from UniProt on Jan 31, 2014; 7802 entries) [64].

### ATP assay

Yeast cultures at the late-log phase were pelleted, and the cell pellets were washed once with PBS and resuspended in 100 μl of 5% trichloroacetic acid (TCA). Cells were vortexed and incubated for 15 min at room temperature. Subsequently, cell debris were pelleted and supernatants were collected and diluted 1:1000 before ATP measurements, which were done using ENLITEN® ATP Assay System Bioluminescence Detection Kits (Promega, WI) according to the manufacturer's instructions.

### The fluorescent microscopy

The morphology of mitochondria was investigated by fluorescent microscopy. Cells were harvested at the late-log phase and washed with PBS and stained with MitoTracker® Green FM (Life Technology, CA) at 500 nM for 30 min according to the manufacturer's instructions. Ten μl of cell suspension was spotted onto a microscopic slide and observed under a fluorescence microscope (Zeiss Axio Imager Z2; Carl Zeiss, Inc., NY).

### Oxygen consumption rate assay

Oxygen consumption rates were measured under a Seahorse instrument (Seahorse Bioscience, MA) according to the manufacturer's instructions. Cells were incubated overnight in either YPD medium for knockout strains or SC-ura medium with 2% galactose for transformed strains and diluted to OD_600_ 0.1 in fresh SC-ura media containing 2% glucose. Diluted cells were used to seed XF96 plates and incubated for 60 min at 30°C. The Seahorse sensor cartridge was rehydrated overnight following the manufacturer's instructions. XF96 culture plates and sensor cartridge were mated and placed in the Seahorse instrument after the initial check-up. Three measurements were taken for the basal reading and averaged. The mean value of the three readings across the 1-min span was calculated for each well, and at least twelve wells were assayed for each strain.

### Mitochondrial membrane potential assay

The mitochondrial inner membrane potential (Δψm) was determined by staining with the membrane-permeable lipophilic cationic fluorochrome JC-1 (BD Biosciences, NJ) according to the manufacturer's instructions. One hundred μg of isolated mitochondria from each yeast strain was loaded onto a 96-well plate and were stained JC-1. The fluorescence intensity at the emission wavelength of 530 (green) or 590 nm (red) was measured under the SpectraMax Plus 384 Microplate Reader (Molecular Devices, CA) and the ratio of the red to green fluorescence intensity was calculated.

### Mitochondrial fusion assay

The mitochondrial fusion assay was performed as described [36]. Briefly, the sequence (N-GFP) encoding an N-terminal mitochondrion-targeting sequence (MTS), the first 157 amino acids of GFP (amino acids 1–157), and a C-terminal leucine zipper, was PCR-amplified using the pVT100U-mtGFP yeast plasmid [65] as a template with the following primers: N-GFP-F (5′-CCCGGGTACCAGATCTATGA GTAAAGGAGAAGAAC-3′) and N-GFP-R (5′-CCGCTCGAGTTGGGCCAATTCCTTTTTTAAAG CCTGTAATTCCCACTTTAATTGGGCTAATTCCTTT TTATTAGCTTGTAATTCCTTTTTTAAAGCACCGGAT CCAGATCCACCCTTTTGTTTGTCTGCCATGAT-3′) (Table S2). The sequence (C-GFP) encoding the N-terminal MTS, the amino acids 158–238 of GFP, and leucine zipper, was PCR-amplified using the pVT100U-mtGFP plasmid as a template and the following primers: C-GFP-F (5′-GGGGTACCGAGCAGTTAGAAAAGAAGTTACA AGCTTTGGAAAAGAAATTGGCACAATTAGAATGG AAGAATCAAGCCTTGGAAAAGAAATTGGCACAA GGTGGATCTGGTAATGGAATCAAAGTTAACTT-3′) and C-GFP-R (5′-CCGCTCGAGGAATTCTTATTTGTATAGTTC-3′) (Table S2). The PCR-amplified DNA fragments were subcloned into the KpnI/XhoI sites of ADH promoter-containing pVT100U to create pVT100U-N-GFP and pVT100U-C-GFP, respectively.

Each of the above vectors was transformed into JK93d WT cell and mitochondria were isolated from each strain harboring either pVT100U-N-GFP or pVT100U-C-GFP. *In vitro* mitochondrial fusion was performed as described previously [66]. Isolated mitochondria were incubated on ice for 20 min and centrifuged at 10,000 g for 2 min. The pellets were suspended in stage 1 buffer (20 mM PIPES-KOH at pH 6.8, 150 mM KOAc, 5 mM Mg(OAc)_2_ and 0.6 M sorbitol) and incubated at 22°C for 20 min. The samples were then centrifuged at 10,000 g for 2 min at 4°C. The pellets were suspended in stage 2 buffer (the stage 1 buffer plus 0.2 mg/mL creatine phosphokinase, 40 mM creatine phosphate, 1 mM ATP and 1 mM GTP) and were incubated at 22°C in the presence of a sphingolipid or FCCP (Sigma, MO) that was used as a mitochondrial fusion inhibitor at a final concentration of 100 μM. The fluorescence intensity at the emission wavelength of 530 (green) was measured under the SpectraMax Plus 384 Microplate Reader (Molecular Devices, CA).

### Quantitative levels of mtDNA

The relative copy numbers of mtDNA were determined using an RT-qPCR strategy as described [67]. Yeast cells were grown in 5 ml of SC-ura medium, and yeast cultures were harvested by centrifugation at late log phase. Total cellular nucleic acids were isolated as described [68] and treated with DNase-free RNase A (10 μg/ml) at 37°C for 1.5 h. RNA-free nuclear DNA and mtDNA were subjected to qPCR, which was run in a standard 25 μl SYBR Green reaction: 12.5 μl of iQ SYBR Green Supermix (Bio-Rad, CA), 10 μl of diluted template, and 1.25 μl of each nuclear gene primer (ACT1 forward and reverse primers, in Table S2) or each mtDNA primer (COX1 forward and reverse primers, in Table S2). The difference [Δ*C*_t=_ *C*_t_ (*COX1*) -*C*_t_ (*ACT1*)] in the average Ct values of COX1 and ACT1 was determined to represent the relative mtDNA copy number of each strain.

### Protein expression analysis

Protein expression was assessed by Western blotting analyses using an appropriate primary antibody and secondary antibody.

### Statistical analysis

Data are presented as the mean ± SD and were compared by either Student's t-test, one-way or two-way ANOVA (ANanlysis Of VAriance) with post hoc Tukey HSD (Honestly Significant Difference) using GraphPad Prism (La Jolla, CA). Values of p<0.05 were considered significant and marked with an asterisk (*).

## SUPPLEMENTARY MATERIAL FIGURES AND TABLES


