# Supplementary material for: Aging-related elevation of sphingoid bases shortens yeast chronological life span by compromising mitochondrial function
Source: Oncotarget. 2016 Mar 19;7(16):21124–44. doi: 10.18632/oncotarget.8195 (PMC5008273; doi:10.18632/oncotarget.8195)
Supplement: Supplementary file 1 [file oncotarget-07-21124-s001.pdf]

Aging-related elevation of sphingoid bases shortens yeast chronological life span by compromising mitochondrial function

Supplementary Material

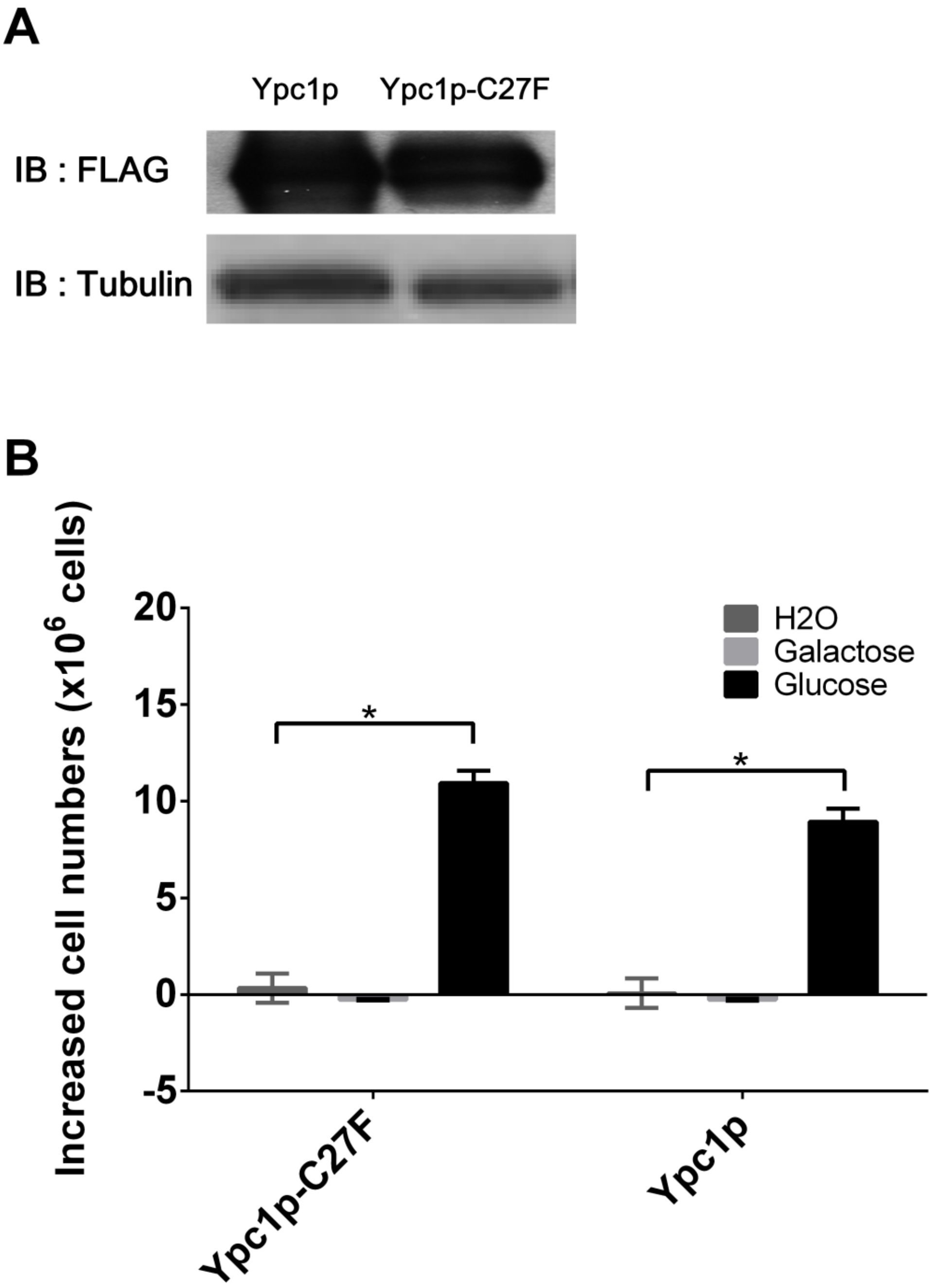

Figure S1. Gal1Δ overexpressing either Ypc1p or Ypc1p-C27F. A, Western blot analyses of Ypc1p and Ypc1p-C27F. Gal1Δ cells expressing the FLAG-tagged Ypc1p or its mutant, Ypc1p-C27F, were analyzed by Western blot analyses using anti-FLAG antibody as described in Materials and Methods. **B**, Cell numbers were counted 24 hr after addition of either H_2O, glucose or galactose to verify if galactose can induce the cell growth in Gal1 Δ. Data represent mean of ± SD; n=3. *$p<0.05$.

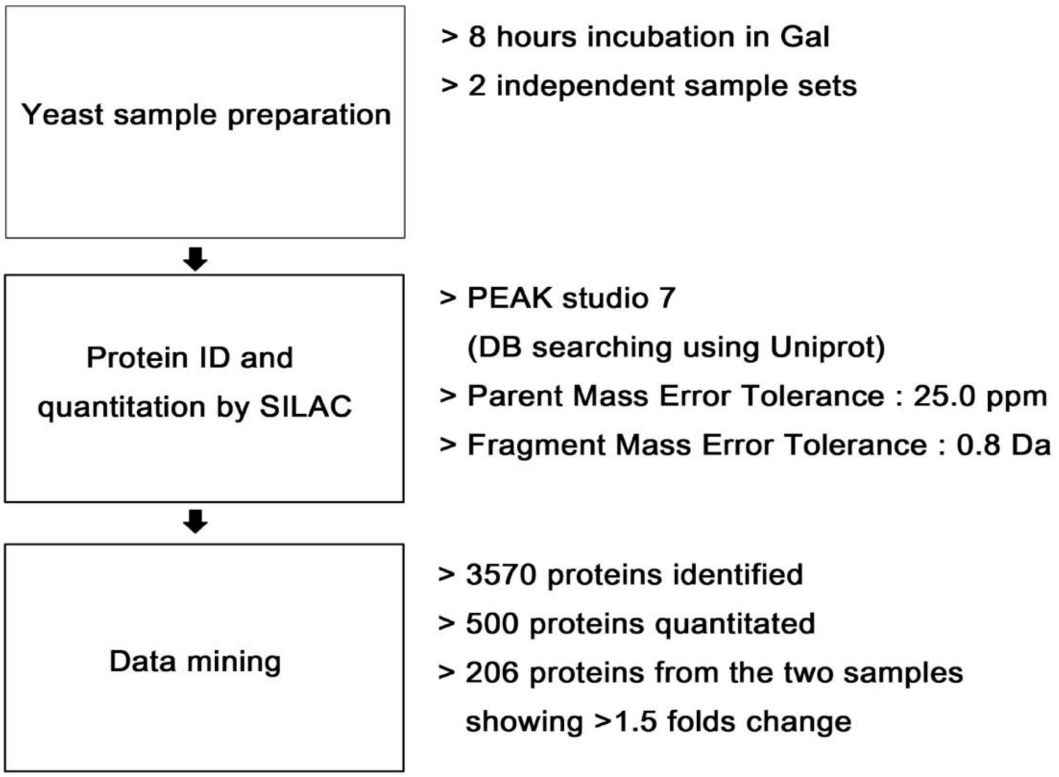

Figure S2 Workflow of SILAC.

Table S1. Strains used in this study

Strain	Genotype	Relevant gene or description	Source
**JK93d**	MATα leu2–3,112 ura3–52 rme1 trp1 his4	Parental strain	This study
**Ydc1Δ**	Isogenic to JK93d, Δ087::UraBbrP1	Δ087=DHcer	[7]
**Ypc1Δ**	Isogenic to JK93d, Δ183::G418	Δ183=PHcer	[7]
**Ydc1ΔYpc1Δ**	Isogenic to JK93d, Δ183::G418 Δ087::UraBbrP1	Δ183=PHcer Δ087=DHcer	[7]
**Lcb4ΔLcb5Δ**	Isogenic to JK93d, lcb4-1 lcb5-1		[39]
**YAG6B**	MATa his3 leu2 met15 ura3 YHR018c::kanMX4 YIR034c::kanMX4	SILAC strain	A kind gift from Dr. Nils Faergeman [31]
**Yi9**	Isogenic to YAG6B, pYES2::FLAG	Empty vector	This study
**Yi10**	Isogenic to YAG6B pYES2::YPC1	Ypc1p overexpression	This study
**Yi11**	Isogenic to YAG6B pYES2::YPC1^{C27F}	Ypc1p-C27F overexpression	This study
**W303-1A**	MATa leu2-3,112 trp1-1 can1-100 ura3-1 ade2-1 his3-11,15		A kind gift from Dr. Ales Vancura [69]
**Msn2ΔMsn4Δ**	Isogenic to W303-1A, msn2::HIS3 msn4::URA3		A kind gift from Dr. Ales Vancura [69]
**BY4742**	MATα his3-1 leu2-0 lys2-0 ura3-0		[70]
**Msn2Δ**	Isogenic to BY4742, msn2::kanMX4		[70]
**Msn4Δ**	Isogenic to BY4742, msn4::kanMX4		[70]

Table S2. Primers used in this study

Targets	Primers	Sequences (5'>3')
**ACT1**	Forward	GTATGTGTAAAGCCGGTTTTG
	Reverse	CATGATACCTTGGTGTCTTGG
**COX1**	Forward	CTACAGATACAGCATTTCCAAGA
	Reverse	GTGCCTGAATAGATGATAATGGT
**YDC1**	Forward	GAATGGGGTTCTCGCTGGTT
	Reverse	TGGTCGCATACAGCATTGGT
**YPC1**	Forward	ATTGGCTTCGGGTACGGTTT
	Reverse	CCTCTTTGGCCTCGCATACT
**LAG1**	Forward	TTACTCCCTTTGTGTTCGGCTT
	Reverse	TCACGACATGGCGCAGATAG
**LAC1**	Forward	TGCTAAAAGGCGTATGCAGAGA
	Reverse	TCAAGGGAGCTATCCAGGCA
**LCB4**	Forward	CTCAGCTACCTTGCGGTTCA
	Reverse	TTTGACAAGGCACAGAGCGG
**LCB5**	Forward	ATCGGAAACATCGACAACGGA
	Reverse	TCTGGCATCCAGAATTCGTCC
**YSR2**	Forward	GCCACGCACAAAGAAGGTG
	Reverse	AGGGAAAATAGGACGGGGCT
**YSR3**	Forward	ATCTGGGTGGTGCATGCTTT
	Reverse	ACAAGCCCCATGCTACACTC
**DPL1**	Forward	CTGCACATGCTGGGTTTGAC
	Reverse	GAGCGGAACCGACCAGTAAA
**N-GFP**	Forward	CCCGGGTACCAGATCTATGAGTAAAGGAGAAGAAC
	Reverse	CCGCTCGAGTTGGGCCAATTCCTTTTTTAAAGCCTGTAATTCCCACTTTAATTGGGCTAATTCCTTTTTATTAGCTTGTAATTCCTTTTTTAAAGCACCGGATCCAGATCCACCCTTTTGTTTGTCTGCCATGAT
**C-GFP**	Forward	GGGGTACCGAGCAGTTAGAAAAGAAGTTACAAGCTTTGGAAAAGAAATTGGCACAATTAGAATGGAAGAATCAAGCCTTGGAAAAGAAATTGGCACAAGGTGGATCTGGTAATGGAATCAAAGTTAACTT

Reverse	CCGCTCGAGGAATTCTTATTTGTATAGTTC

Table S3. STREs in promoter regions of the genes of yeast sphingolipid-metabolizing enzymes.

Genes	No. of STREs in the promoter region	Distance between two STREs (nt)
YDC1	1	N/A
YPC1	3	85, 185
LAG1	2	462 nt
LAC1	1	N/A
DPL1	1	N/A
YSR2	1	N/A
YSR3	0	N/A
LCB4	2	196
LCB5	0	N/A

Table S4. Expression ratio of proteins in yeast cells in response to YPC1 overexpression (SILAC). (H, Ypc1p-overexpression. L, Ypc1p-C27F overexpression.)

Accession	Gene	Description	Average ratio (H:L)
P15992	HSP26	Heat shock protein 26	5.90
P09733	TUB1	Tubulin alpha-1 chain	4.08
P38298	YPC1	Alkaline ceramidase YPC1	3.94
P53228	NQM1	Transaldolase NQM1	3.90
P32368	SAC1	Phosphoinositide phosphatase SAC1	3.20
P29952	PMI40	Mannose-6-phosphate isomerase	3.15
Q00055	GPD1	Glycerol-3-phosphate dehydrogenase [NAD(+)] 1	3.14
P00330	ADH1	Alcohol dehydrogenase 1	2.84
Q04432	HSP31	Probable chaperone protein HSP31	2.63
P32602	SEC17	Alpha-soluble NSF attachment protein	2.63
P14832	CPR1	Peptidyl-prolyl cis-trans isomerase	2.59
P37291	SHM2	Serine hydroxymethyltransferase cytosolic	2.55
P07264	LEU1	3-isopropylmalate dehydratase	2.47
P32356	NTH1	Neutral trehalase	2.43
P06780	RHO1	GTP-binding protein RHO1	2.36
Q04728	ARG7	Arginine biosynthesis bifunctional protein ArgJ	2.33
Q12154	GET3	ATPase GET3	2.33
Q06151	DCS1	m7GpppX diphosphatase	2.32
P38067	UGA2	Succinate-semialdehyde dehydrogenase [NADP(+)]	2.31
P38013	AHP1	Peroxiredoxin type-2	2.29
Q12207	NCE102	Non-classical export protein 2	2.26
Q12335	PST2	Protoplast secreted protein 2	2.25
P13586	PMR1	Calcium-transporting ATPase 1	2.25

P54838	DAK1	Dihydroxyacetone kinase 1	2.21
P00950	GPM1	Phosphoglycerate mutase 1	2.16
P06169	PDC1	Pyruvate decarboxylase isozyme 1	2.16
Q12019	MDN1	Midasin	2.14
Q03558	OYE2	NADPH dehydrogenase 2	2.06
P14904	LAP4	Vacuolar aminopeptidase 1	2.05
P00358	TDH2	Glyceraldehyde-3-phosphate dehydrogenase 2	2.04
Q01976	YSA1	ADP-ribose pyrophosphatase	2.03
P25293	NAP1	Nucleosome assembly protein	1.99
Q12458	YPR1	Putative reductase 1	1.97
P38079	YRO2	Protein YRO2	1.97
P06208	LEU4	2-isopropylmalate synthase	1.95
P04385	GAL1	Galactokinase	1.94
P07267	PEP4	Saccharopepsin	1.93
P06738	GPH1	Glycogen phosphorylase	1.91
P38715	GRE3	NADPH-dependent aldose reductase GRE3	1.90
P54783	ALO1	D-arabinono-1 4-lactone oxidase	1.89
P46367	ALD4	Potassium-activated aldehyde dehydrogenase	1.87
P47018	MTC1	Maintenance of telomere capping protein 1	1.87
P12709	PGI1	Glucose-6-phosphate isomerase	1.86
P37012	PGM2	Phosphoglucomutase-2	1.85
P47143	ADO1	Adenosine kinase	1.85
P80210	ADE12	Adenylosuccinate synthetase	1.84
P41338	ERG10	Acetyl-CoA acetyltransferase	1.84
P38972	ADE6	Phosphoribosylformylglycinamidine synthase	1.83
P17255	VMA1	V-type proton ATPase catalytic subunit A	1.81

P39958	GDI1	Rab GDP-dissociation inhibitor	1.81
Q06142	KAP95	Importin subunit beta-1	1.81
P00549	CDC19	Pyruvate kinase 1	1.81
P38427	TSL1	Trehalose synthase complex regulatory subunit TSL1	1.81
Q07551	YDL124W	NADPH-dependent alpha-keto amide reductase G	1.81
P22147	XRN1	5'-3' exoribonuclease 1	1.81
P16862	PFK2	6-phosphofructokinase subunit beta	1.80
P04802	DPS1	Aspartate--tRNA ligase cytoplasmic	1.79
P00942	TPI1	Triosephosphate isomerase	1.77
P08524	ERG20	Farnesyl pyrophosphate synthase	1.77
P31539	HSP104	Heat shock protein 104	1.77
P38911	FPR3	FK506-binding nuclear protein	1.76
P41921	GLR1	Glutathione reductase	1.74
P11154	PYC1	Pyruvate carboxylase 1	1.74
Q12074	SPE3	Spermidine synthase	1.73
P14540	FBA1	Fructose-bisphosphate aldolase	1.73
P49089	ASN1	Asparagine synthetase [glutamine-hydrolyzing] 1	1.73
P25694	CDC48	Cell division control protein 48	1.71
P38625	GUA1	GMP synthase [glutamine-hydrolyzing]	1.71
P32379	PUP2	Proteasome subunit alpha type-5	1.71
P34227	PRX1	Mitochondrial peroxiredoxin PRX1	1.70
P00729	PRC1	Carboxypeptidase Y	1.69
P14742	GFA1	Glutamine--fructose-6-phosphate aminotransferase	1.68
P15496	IDI1	Isopentenyl-diphosphate Delta-isomerase	1.68
Q99383	HRP1	Nuclear polyadenylated RNA-binding protein 4	1.67
P32589	SSE1	Heat shock protein homolog SSE1	1.66

Q05016	YMR226C	Uncharacterized oxidoreductase YMR226C G	1.66
P38993	FET3	Iron transport multicopper oxidase FET3	1.65
P23724	PRE7	Proteasome subunit beta type-6	1.64
P36114	YKR018C	Mitochondrial outer membrane protein YKR018C G	1.64
P15891	ABP1	Actin-binding protein	1.64
P04840	POR1	Mitochondrial outer membrane protein porin 1	1.64
P32527	ZUO1	Zuotin	1.62
P40303	PRE6	Proteasome subunit alpha type-4	1.61
P31412	VMA5	V-type proton ATPase subunit C	1.60
P15703	BGL2	Glucan 1 3-beta-glucosidase	1.60
Q12118	SGT2	Small glutamine-rich tetratricopeptide repeat-containing protein 2	1.60
P40150	SSB2	Heat shock protein SSB2	1.60
P46956	PHO86	Inorganic phosphate transporter PHO86	1.59
P53173	ERV14	ER-derived vesicles protein ERV14	1.58
P32486	KRE6	Beta-glucan synthesis-associated protein KRE6	1.58
P53278	YGR130C	Uncharacterized protein YGR130C G	1.58
P14906	SEC63	Protein translocation protein SEC63	1.57
Q05359	ERP1	Protein ERP1	1.57
P29547	CAM1	Elongation factor 1-gamma 1	1.56
P40010	NUG1	Nuclear GTP-binding protein NUG1	1.55
P41811	SEC27	Coatomer subunit beta'	1.55
P05030	PMA1	Plasma membrane ATPase 1	1.55
Q12754	RRP12	Ribosomal RNA-processing protein 12	1.55
Q03048	COF1	Cofilin	1.54
P39730	FUN12	Eukaryotic translation initiation factor 5B	1.54
P16861	PFK1	6-phosphofructokinase subunit alpha	1.54

P39076	CCT2	T-complex protein 1 subunit beta	1.53
P04807	HXK2	Hexokinase-2	1.53
Q12532	TAE2	Translation-associated element 2	1.53
P08431	GAL7	Galactose-1-phosphate uridylyltransferase	1.52
Q08972	NEW1	[NU+] prion formation protein 1	1.51
Q01939	RPT6	26S protease regulatory subunit 8 homolog	1.51
P54113	ADE16	Bifunctional purine biosynthesis protein ADE16	1.51
P15705	STI1	Heat shock protein STI1	1.51
P17709	GLK1	Glucokinase-1	1.50
Q05567	DPL1	Sphingosine-1-phosphate lyase	1.50
P22515	UBA1	Ubiquitin-activating enzyme E1 1	1.50
P32497	NIP1	Eukaryotic translation initiation factor 3 subunit C	1.50
P07170	ADK1	Adenylate kinase	1.50
P22023	KRE5	Killer toxin-resistance protein 5	1.49
P54885	PRO2	Gamma-glutamyl phosphate reductase	1.49
P32835	GSP1	GTP-binding nuclear protein GSP1/CNR1	1.49
P32836	GSP2	GTP-binding nuclear protein GSP2/CNR2	1.49
P39993	GEA2	ARF guanine-nucleotide exchange factor 2	1.48
P22137	CHC1	Clathrin heavy chain	1.47
Q07478	SUB2	ATP-dependent RNA helicase SUB2	1.47
P25491	YDJ1	Mitochondrial protein import protein MAS5	1.47
P00445	SOD1	Superoxide dismutase [Cu-Zn]	1.46
P35732	DEF1	RNA polymerase II degradation factor 1	1.45
P24031	PHO3	Constitutive acid phosphatase	1.44
P31383	TPD3	Protein phosphatase PP2A regulatory subunit A	1.44
P19146	ARF2	ADP-ribosylation factor 2	1.44

P39683	NPT1	Nicotinate phosphoribosyltransferase	1.43
P40024	ARB1	ABC transporter ATP-binding protein ARB1	1.43
P34760	TSA1	Peroxiredoxin TSA1	1.43
Q02932	KAP120	Importin beta-like protein KAP120	1.43
P15303	SEC23	Protein transport protein SEC23	1.43
P22146	GAS1	1 3-beta-glucanosyltransferase GAS1	1.43
P38707	DED81	Asparagine--tRNA ligase cytoplasmic	1.43
P20606	SAR1	Small COPII coat GTPase SAR1	1.43
P16603	NCP1	NADPH--cytochrome P450 reductase	1.43
P47176	BAT2	Branched-chain-amino-acid aminotransferase cytosolic	1.43
P40474	QDR2	Quinidine resistance protein 2	1.42
P47037	SMC3	Structural maintenance of chromosomes protein 3	1.42
Q03940	RVB1	RuvB-like protein 1	1.42
P32352	ERG2	C-8 sterol isomerase	1.42
P36008	TEF4	Elongation factor 1-gamma 2	1.41
P40302	PRE5	Proteasome subunit alpha type-6	1.41
P27692	SPT5	Transcription elongation factor SPT5	1.41
P19524	MYO2	Myosin-2	1.41
P52593	NUP188	Nucleoporin NUP188	1.40
P25294	SIS1	Protein SIS1	1.40
Q03103	ERO1	Endoplasmic oxidoreductin-1	1.40
P32471	EFB1	Elongation factor 1-beta	1.40
P38623	RCK2	Serine/threonine-protein kinase RCK2	1.40
P40069	KAP123	Importin subunit beta-4	1.40
P19097	FAS2	Fatty acid synthase subunit alpha	1.39
P46680	AIP1	Actin-interacting protein 1	1.38

P07342	ILV2	Acetolactate synthase catalytic subunit mitochondrial	1.38
P23615	SPT6	Transcription elongation factor SPT6	1.38
P0CX25	RPL43A	60S ribosomal protein L43-A	1.37
P33401	PGM1	Phosphoglucomutase-1	1.37
P07703	RPC40	DNA-directed RNA polymerases I and III subunit RPAC1	1.37
P17555	SRV2	Adenylyl cyclase-associated protein	1.37
P32565	RPN2	26S proteasome regulatory subunit RPN2	1.36
P32337	PSE1	Importin subunit beta-3	1.36
P0CX55	RPS18A	40S ribosomal protein S18-A	1.36
Q99190	TSC13	Very-long-chain enoyl-CoA reductase	1.36
P23254	TKL1	Transketolase 1	1.36
P10081	TIF1	ATP-dependent RNA helicase eIF4A	1.36
Q02642	EGD1	Nascent polypeptide-associated complex subunit beta-1	1.35
P53691	CPR6	Peptidyl-prolyl cis-trans isomerase CPR6	1.35
P10080	SBP1	Single-stranded nucleic acid-binding protein	1.35
P28272	URA1	Dihydroorotate dehydrogenase (fumarate)	1.35
Q00955	ACC1	Acetyl-CoA carboxylase	1.35
P53914	KRE33	UPF0202 protein KRE33	1.34
Q02486	ABF2	ARS-binding factor 2 mitochondrial	1.34
Q04951	SCW10	Probable family 17 glucosidase SCW10	1.34
P36047	SDS22	Protein phosphatase 1 regulatory subunit SDS22	1.33
Q07896	NOC3	Nucleolar complex-associated protein 3	1.33
Q05905	HRI1	Protein HRI1	1.33
P41940	PSA1	Mannose-1-phosphate guanyltransferase	1.32
P33307	CSE1	Importin alpha re-exporter	1.32
P02829	HSP82	ATP-dependent molecular chaperone HSP82	1.32

P26637	CDC60	Leucine--tRNA ligase cytoplasmic	1.31
P29704	ERG9	Squalene synthase	1.31
Q03161	YMR099C	Glucose-6-phosphate 1-epimerase G	1.31
P07263	HTS1	Histidine--tRNA ligase mitochondrial	1.31
P27614	CPS1	Carboxypeptidase S	1.30
P25375	PRD1	Saccharolysin	1.30
Q12250	RPN5	26S proteasome regulatory subunit RPN5	1.30
P10592	SSA2	Heat shock protein SSA2	1.30
P15454	GUK1	Guanylate kinase	1.29
P11745	RNA1	Ran GTPase-activating protein 1	1.29
Q03690	CLU1	Clustered mitochondria protein 1	1.29
P15108	HSC82	ATP-dependent molecular chaperone HSC82	1.28
P04397	GAL10	Bifunctional protein GAL10	1.28
P40495	LYS12	Homoisocitrate dehydrogenase mitochondrial	1.28
P16521	YEF3	Elongation factor 3A	1.28
P00817	IPP1	Inorganic pyrophosphatase	1.28
P06105	SCP160	Protein SCP160	1.27
P26321	RPL5	60S ribosomal protein L5	1.27
P00925	ENO2	Enolase 2	1.27
P25087	ERG6	Sterol 24-C-methyltransferase	1.27
P02994	TEF1	Elongation factor 1-alpha	1.27
P02407	RPS17A	40S ribosomal protein S17-A	1.26
P38934	BFR1	Nuclear segregation protein BFR1	1.26
P15646	NOP1	rRNA 2'-O-methyltransferase fibrillarin	1.26
P35691	TMA19	Translationally-controlled tumor protein homolog	1.26
P36015	YKT6	Synaptobrevin homolog YKT6	1.26

P07260	CDC33	Eukaryotic translation initiation factor 4E	1.25
P36016	LHS1	Heat shock protein 70 homolog LHS1	1.25
P32324	EFT1	Elongation factor 2	1.25
P46654	RPS0B	40S ribosomal protein S0-B	1.25
P11484	SSB1	Heat shock protein SSB1	1.25
P41807	VMA13	V-type proton ATPase subunit H	1.25
P53731	ARC35	Actin-related protein 2/3 complex subunit 2	1.24
P33775	PMT1	Dolichyl-phosphate-mannose--protein mannosyltransferase 1	1.23
P32473	PDB1	Pyruvate dehydrogenase E1 component subunit beta	1.23
P15180	KRS1	Lysine--tRNA ligase cytoplasmic	1.23
P09436	ILS1	Isoleucine--tRNA ligase cytoplasmic	1.22
P47079	CCT8	T-complex protein 1 subunit theta	1.22
Q12117	MRH1	Protein MRH1	1.22
P0CX36	RPS4B	40S ribosomal protein S4-B	1.22
P05759	RPS31	Ubiquitin-40S ribosomal protein S31	1.22
P53064	RTF1	RNA polymerase-associated protein RTF1	1.21
P29509	TRR1	Thioredoxin reductase 1	1.21
Q12466	TCB1	Tricalbin-1	1.20
P0CX39	RPS8A	40S ribosomal protein S8-A	1.20
Q03532	HAS1	ATP-dependent RNA helicase HAS1	1.19
P34160	STO1	Nuclear cap-binding protein complex subunit 1	1.19
Q08179	MDM38	Mitochondrial distribution and morphology protein 38	1.19
P38817	GGA2	ADP-ribosylation factor-binding protein GGA2	1.19
P05737	RPL7A	60S ribosomal protein L7-A	1.18
P36060	MCR1	NADH-cytochrome b5 reductase 2	1.18
P52918	MSN5	Protein MSN5	1.18

P07284	SES1	Serine--tRNA ligase cytoplasmic	1.18
P0CX54	RPL12B	60S ribosomal protein L12-B	1.18
P14020	DPM1	Dolichol-phosphate mannosyltransferase	1.17
P31373	CYS3	Cystathionine gamma-lyase	1.17
P20967	KGD1	2-oxoglutarate dehydrogenase mitochondrial	1.17
Q12447	PAA1	Polyamine N-acetyltransferase 1	1.17
P27476	NSR1	Nuclear localization sequence-binding protein	1.17
Q08977	YPL260W	UPF0662 protein YPL260W G	1.17
P0CX83	RPL19B	60S ribosomal protein L19-B	1.17
P41805	RPL10	60S ribosomal protein L10	1.16
P39077	CCT3	T-complex protein 1 subunit gamma	1.15
P23248	RPS1B	40S ribosomal protein S1-B	1.15
P0CX48	RPS11B	40S ribosomal protein S11-B	1.15
P26785	RPL16B	60S ribosomal protein L16-B	1.14
P0CX42	RPL23B	60S ribosomal protein L23-B	1.13
P28777	ARO2	Chorismate synthase	1.12
P39078	CCT4	T-complex protein 1 subunit delta	1.12
P05317	RPP0	60S acidic ribosomal protein P0	1.12
P09457	ATP5	ATP synthase subunit 5 mitochondrial	1.12
P15019	TAL1	Transaldolase	1.11
P0CX52	RPS16B	40S ribosomal protein S16-B	1.11
P33442	RPS1A	40S ribosomal protein S1-A	1.10
Q02753	PL21A	60S ribosomal protein L21-A	1.09
P33322	CBF5	H/ACA ribonucleoprotein complex subunit 4	1.09
P05626	ATP4	ATP synthase subunit 4 mitochondrial	1.09
P40212	RPL13B	60S ribosomal protein L13-B	1.08

P38328	ARC40	Actin-related protein 2/3 complex subunit 1	1.07
P0CX46	RPL2B	60S ribosomal protein L2-B	1.06
P38687	SRP68	Signal recognition particle subunit SRP68	1.06
Q06252	YLR179C	Uncharacterized protein YLR179C G	1.06
P28241	IDH2	Isocitrate dehydrogenase [NAD] subunit 2 mitochondrial	1.06
P0CX49	RPL18A	60S ribosomal protein L18-A	1.05
P07280	RPS19A	40S ribosomal protein S19-A	1.05
P26783	RPS5	40S ribosomal protein S5	1.03
P0CS90	SSC1	Heat shock protein SSC1 mitochondrial	0.96
P14126	RPL3	60S ribosomal protein L3	0.96
P25443	RPS2	40S ribosomal protein S2	0.96
P0CX29	RPS23A	40S ribosomal protein S23-A	0.94
P15873	POL30	Proliferating cell nuclear antigen	0.93
P18239	PET9	ADP ATP carrier protein 2	0.93
P02406	RPL28	60S ribosomal protein L28	0.92
P05373	HEM2	Delta-aminolevulinic acid dehydratase	0.92
P42943	CCT7	T-complex protein 1 subunit eta	0.89
P0C0W1	RPS22A	40S ribosomal protein S22-A	0.87
Q3E7Y3	RPS22B	40S ribosomal protein S22-B	0.87
P00128	QCR7	Cytochrome b-c1 complex subunit 7	0.87
P32191	GUT2	Glycerol-3-phosphate dehydrogenase mitochondrial	0.86
P19414	ACO1	Aconitate hydratase mitochondrial	0.86
Q00711	SDH1	Succinate dehydrogenase flavoprotein subunit mitochondrial	0.85
Q12680	GLT1	Glutamate synthase [NADH]	0.84
P40215	NDE1	External NADH-ubiquinone oxidoreductase 1	0.83

		mitochondrial	
P36010	YNK1	Nucleoside diphosphate kinase	0.83
P05375	OPI3	Phosphatidyl-N-methylethanolamine N-methyltransferase	0.80
P07143	CYT1	Cytochrome c1 heme protein mitochondrial	0.78
P23776	EXG1	Glucan 1 3-beta-glucosidase I/II	0.78
P37303	GLY1	Low specificity L-threonine aldolase	0.76
P47120	LIA1	Deoxyhypusine hydroxylase	0.76
P10659	SAM1	S-adenosylmethionine synthase 1	0.69
P25555	GBP2	Single-strand telomeric DNA-binding protein GBP2	0.65
P21801	SDH2	Succinate dehydrogenase iron-sulfur subunit mitochondrial	0.58
P39105	PLB1	Lysophospholipase 1	0.51
P32340	NDI1	Rotenone-insensitive NADH-ubiquinone oxidoreductase	0.50
P39676	YHB1	Flavohemoprotein	0.45
Q12512	ZPS1	Protein ZPS1	0.45
P32468	CDC12	Cell division control protein 12	0.26
P06106	MET17	Protein MET17	0.18
P32804	ZRT1	Zinc-regulated transporter 1	0.11
